# Influence of ligand encapsulation on cobalt-59 chemical-shift thermometry†
†Electronic supplementary information (ESI) available. See DOI: 10.1039/c9sc01689a


**DOI:** 10.1039/c9sc01689a

**Published:** 2019-06-05

**Authors:** Tyler M. Ozvat, Manuel E. Peña, Joseph M. Zadrozny

**Affiliations:** a Department of Chemistry , Colorado State University , Fort Collins , Colorado 80523 , USA . Email: joe.zadrozny@colostate.edu

## Abstract

Thermometry *via* magnetic resonance imaging (MRI) would provide a powerful noninvasive window into physiological temperature management. Cobalt-59 nuclear spins demonstrate exceptional temperature dependence of their NMR chemical shifts, yet the insight to control this dependence *via* molecular design is lacking. We present the first systematic evidence that encapsulation of this spin system amplifies the temperature sensitivity. We tested the temperature dependence of the ^59^Co chemical shift (Δ*δ*/Δ*T*) in a series of five low-spin cobalt(iii) complexes as a function of increasing encapsulation within the 1st coordination sphere. This study spans from [Co(NH_3_)_6_]Cl_3_, with no interligand connectivity, to a fully encapsulated dinitrosarcophagine (diNOsar) complex, [Co(diNOsar)]Cl_3_. We discovered Δ*δ*/Δ*T* values that span from 1.44(2) ppm °C^–1^ in [Co(NH_3_)_6_]Cl_3_ to 2.04(2) ppm °C^–1^ in [Co(diNOsar)]Cl_3_, the latter among the highest for a molecular complex. The data herein suggest that designing ^59^Co NMR thermometers toward high chemical stability can be coincident with high Δ*δ*/Δ*T*. To better understand this phenomenon, variable-temperature UV-Vis, ^59^Co NMR relaxation, Raman spectroscopic, and variable-solvent investigations were performed. Data from these measurements highlight an unexpected impact of encapsulation – an increasingly dynamic and flexible inner coordination sphere. These results comprise the first systematic studies to reveal insight into the molecular factors that govern Δ*δ*/Δ*T* and provide the first evidence of ^59^Co nuclear-spin control *via* vibrational means.

## Abstract 2

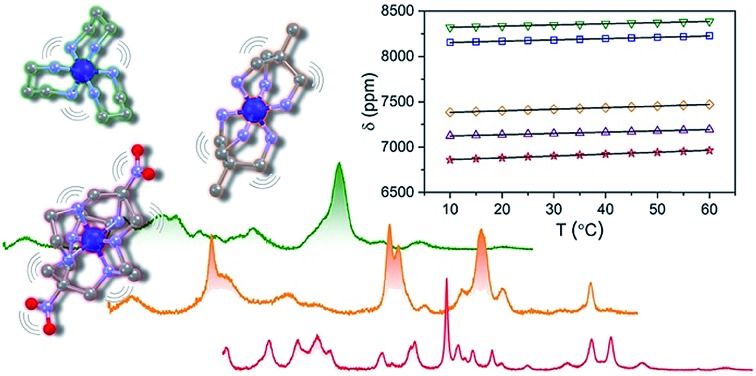
This manuscript details the first investigation of ligand encapsulation on thermometry by cobalt-59 nuclear spins.

## Introduction

The structural flexibilities of metal complexes are key design principles for applications in the areas of reactivity,^1^,^2^ medicine,^3^ photophysical properties,^4^ and magnetic information storage.^5^ Flexibility engenders stimuli-dependent changes in the coordination geometry of a metal, hence impacting d-orbital energies and any properties stemming from electronic structure.^6^ Thus, the control of flexibility is potentially a powerful way for targeting applications for metal complexes. One such application is biomedical thermometry by magnetic resonance imaging (MRI),^7^,^8^ where the temperature-dependent structure of a flexible complex induces highly temperature-dependent spin-Hamiltonian parameters or relaxation times. If this variation could be harnessed to develop an imaging technique, such an application would circumvent many of the challenges associated with invasive thermometry, *e.g.* the point-like nature of the measurement.

**Fig. 1:**
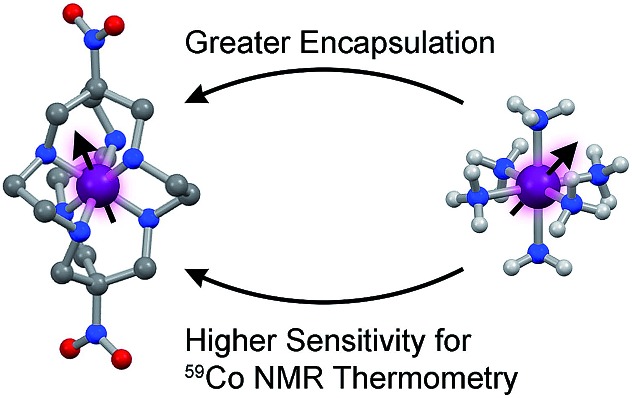
Tested design parameter in this manuscript. Molecular structures of (left) [Co(diNOsar)]^3+^ (diNOsar = dinitrosarcophagine) and (right) [Co(NH_3_)_6_]^3+^ are taken from crystal structures in ref. 23 and 24. Anions and hydrogens omitted for clarity where necessary. Purple, blue, red, grey, and light grey spheres correspond to cobalt, nitrogen, oxygen, carbon, and hydrogen atoms, respectively.

One promising system for such thermometry by magnetic resonance is the cobalt-59 nucleus in low-spin cobalt(iii) complexes. This NMR-active nucleus is 100% naturally abundant, *I* = 7/2, and has a receptivity of *ca.* 30% that of ^1^H. Furthermore, the nucleus displays a wide reported chemical shift (*δ*) window (20 000 ppm) as a result of a paramagnetic contribution to *δ* that is directly tied to the ligand field splitting, *Δ*_o_.^9^–^11^ For complexes that contain cobalt-59 nuclei, changes in solution structure, such as lengthening Co–ligand bonds, can impact *Δ*_o_, imparting changes in *δ*, and providing a mechanism for thermometry. In principle, then, the ^59^Co chemical shift could be used to spatially map temperature through a technique known as chemical shift imaging.^12^–^14^ Initial studies reveal sensitivities (Δ*δ*/Δ*T*) on the order of **1–3** ppm °C^–1^,^15^–^20^ order-of-magnitude upgrades to the possibilities for conventional ^1^H NMR thermometry.^21^,^22^ Hence, these species may be useful to develop as new probes for chemical-shift imaging of temperature.^12^ However, fundamental insight about the factors to govern that sensitivity is lacking (this manuscript explores one factor – encapsulation – as depicted in Fig. 1). As a result, design principles for enhancing Δ*δ*/Δ*T* values are absent, and the true potential of cobalt-59 NMR thermometers for MRI remains unrealized.

**Fig. 2:**
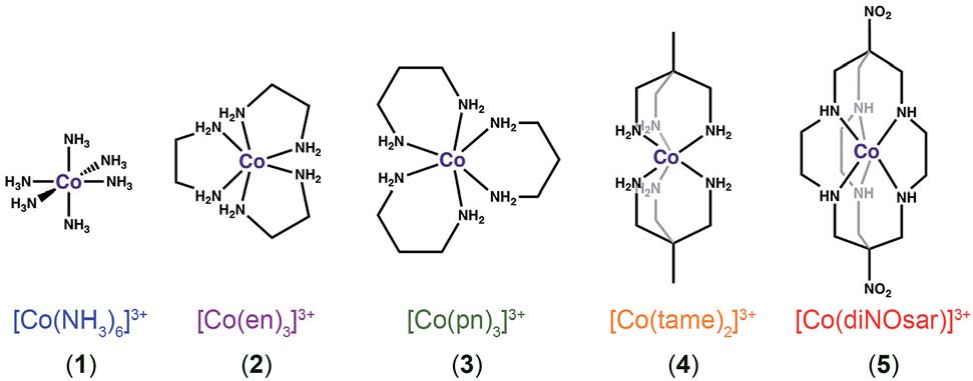
Bond-line representations of complexes **1–5**, the systems studied in this manuscript. Anions and hydrogens bound to carbons are omitted for clarity.

This manuscript details the first systematic exploration of the molecular factors that govern Δ*δ*/Δ*T* in a family of cobalt-59 NMR thermometers (Fig. 1 and 2). As a first step, we sought to explore the role of ligand encapsulation on the temperature sensitivity of the ligand field and cobalt-59 NMR properties. Encapsulation is known to afford enhanced stability for metal complexes *via* the chelate and related macrocyclic effects.^25^ Such stability is an important property for any imaging agent, as release of the metal can both induce toxicity and deactivate the magnetic species being used as a sensor. At the same time, a rigid, encapsulated ion can be readily envisioned to lack the flexibility needed for thermometry *via* structural change. Hence, encapsulation, while affording significant chemical stability, might simultaneously subdue the ability to sense temperature *via* cobalt-59 NMR.

To test this hypothesis, we investigated Δ*δ*/Δ*T* for the ^59^Co nuclei of the low-spin Co(iii) complexes (Fig. 2) [Co(NH_3_)_6_]Cl_3_ (**1**),^26^ [Co(en)_3_]Cl_3_ (**2**, en = ethylenediamine),^27^ [Co(pn)_3_]Cl_3_ (**3**, pn = 1,3-diaminopropane),^28^ [Co(tame)_2_]Cl_3_ (**4**, tame = triaminomethylethane),^29^ [Co(diNOsar)]Cl_3_ (**5**, = dinitrosarcophagine),^23^ and K_3_[Co(CN)_6_], the ^59^Co NMR standard. This series of complexes was selected to enable an investigation of both molecular and electronic structure on Δ*δ*/Δ*T*. First, we hypothesized that the increasing connectivity between the nitrogen donor atoms in **1–5** would engender an increasingly rigid coordination environment and, hence, suppress Δ*δ*/Δ*T*. Thus, we expected that sensitivity to temperature would be compromised in favor of chemical stability. Indeed, the fully-encapsulating sarcophagine scaffold^23^,^25^,^30^ will only surrender its NMR-active Co(iii) ion under harsh conditions – heating in concentrated cyanide solution or acidic media.^31^,^32^ The second investigation enabled by this set of complexes is the test of whether Δ*δ*/Δ*T* directly correlates with *Δ*_o_. The ^59^Co chemical shift is proportional to 1/*Δ*_o_,^10^ hence, *δ* should be more sensitive to tiny fluctuations in *Δ*_o_ at lower *Δ*_o_.^10^ These studies are the first to reveal three key facts about Δ*δ*/Δ*T*. Firstly, in contrast to our expectations, encapsulation enhances Δ*δ*/Δ*T*. That is – the “rigid” ligand frameworks in **5** and **4** induce a stronger temperature-dependence in *Δ*_o_ (and *δ*) than the less-encapsulated species **1–3**. Indeed, variable-temperature UV-Vis and ^59^Co spin-lattice relaxation studies indicate that encapsulation counterintuitively supports higher temperature dependence in the coordination geometry. Second, our studies show that *Δ*_o_ alone does not correlate to the magnitude of Δ*δ*/Δ*T*. Finally, third, Raman spectroscopy studies suggest molecular vibrational lifetimes – prolonged by high interconnectivity among donor atoms – are important factors governing Δ*δ*/Δ*T*. Together, the data highlight a new implication for rigidity in molecular magnetism.

## Results and discussion

**Fig. 3:**
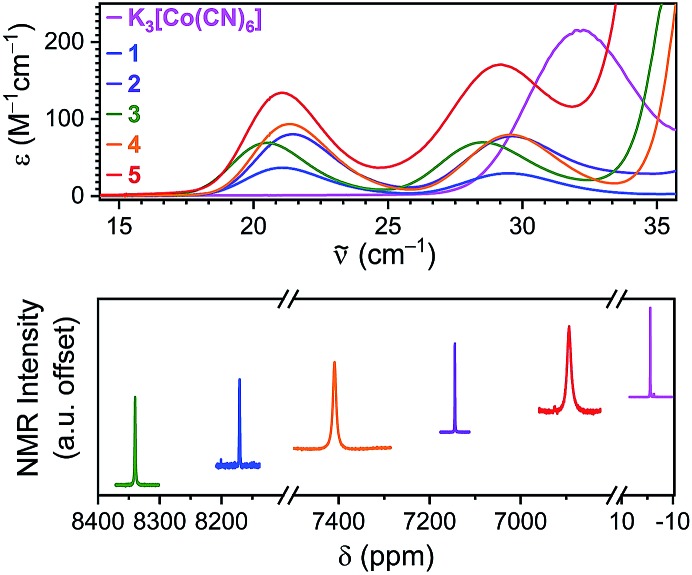
Characterization of Co(iii) electronic structure in **1–5**. Top: electronic absorption (UV-Vis) spectra for **1–5** and K_3_[Co(CN)_6_] in H_2_O at room temperature. The lower-energy peak is the ^1^A_1g_ to ^1^T_1g_ transition while the higher-wavenumber peak is ^1^A_1g_ to ^1^T_2g_. Bottom: 500 MHz ^59^Co NMR spectra for **1–5** in H_2_O at room temperature.

Understanding the temperature sensitivity of the chemical shift requires first establishing the electronic structures of the cobalt(iii) ions in **1–5**. UV-Vis electronic absorption spectra of compounds **1–5** and K_3_[Co(CN)_6_] in H_2_O reproduce reported results for the individual complexes (Fig. 3), wherein the lowest energy peak indicates the ^1^A_1g_ → ^1^T_1g_ transition and the higher energy peak indicates the ^1^A_1g_ → ^1^T_2g_ transition.^10^,^23^ The energies of these two peaks and a Tanabe-Sugano diagram permit quantitation of *Δ*_o_, which increases from **3** (22 376 cm^–1^) to **5** (22 754 cm^–1^) and **1** (23 018 cm^–1^) to **4** (23 276 cm^–1^) to **2** (23 321 cm^–1^) (Table S1†). These values are consistent with literature values for **1–5** and stand in contrast to the strong ligand field of K_3_[Co(CN)_6_] that engenders a *Δ*_o_ of 38 000 cm^–1^.^33^^59^Co resonant frequencies were observed for **1–5** over the range of 6800 to 8400 ppm (referenced to K_3_[Co(CN)_6_]). According to the ^59^Co chemical shifts, the magnitude of *Δ*_o_ increases in the order **3** < **1** < **4** < **2** < **5** < K_3_[Co(CN)_6_]. This order is at odds with the trend obtained from electronic absorption spectroscopy measurements (see Fig. S1†). However, reported correlations between UV-Vis peak position and *δ* are only approximate, not quantitative.^9^,^10^ Nevertheless, these measurements provide (i) two points of reference to test for a correlation between Δ*δ*/Δ*T* and *Δ*_o_, and (ii) the location of the ^59^Co NMR resonances for variable-temperature analyses.

**Fig. 4:**
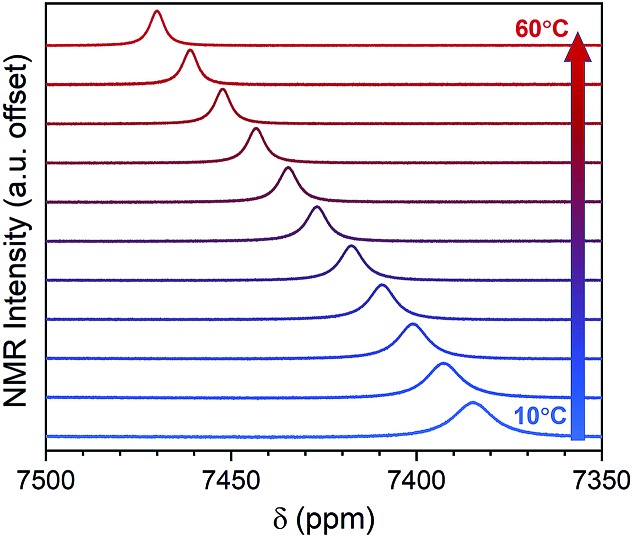
Variable-temperature ^59^Co NMR for **4** at 500 MHz in H_2_O, collected in increments of 5 °C. The system was allowed to equilibrate for at least 5 minutes between each temperature point prior to measurement.

**Fig. 5:**
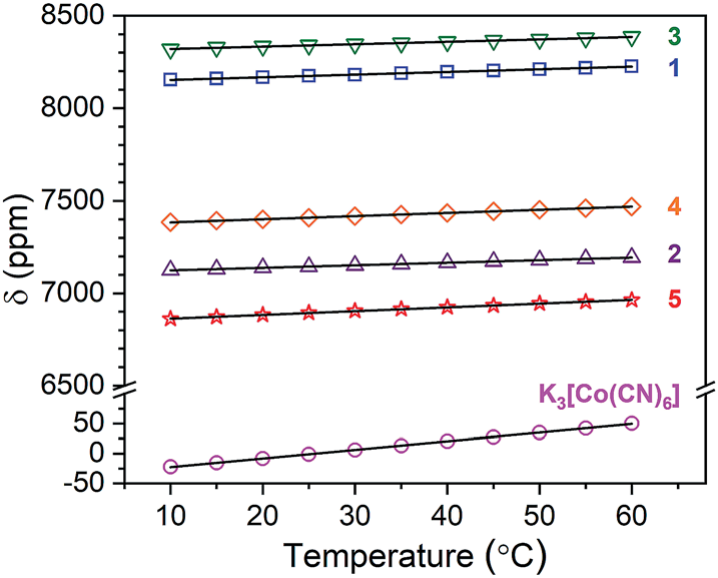
Chemical shift values for **1–5** and K_3_[Co(CN)_6_] as a function of temperature. Solid lines are the result of linear regression – see main text for further details.

Variable-temperature ^59^Co NMR spectra were collected for **1–5** and K_3_[Co(CN)_6_] in H_2_O from 10–60 °C (see Fig. 4, 5 and S2–S7†) to explore the temperature dependence of *δ*. With increasing temperature, peaks for **1–5** and K_3_[Co(CN)_6_] shift downfield to higher *δ*. This temperature-dependent shift of peaks is consistent with varying coordination geometry in solution.^18^,^34^ As temperature is increased, energy is introduced into the vibrational modes of the cobalt complex, expanding M–L bond distances and engendering generally weaker *Δ*_o_.^18^,^35^

Precise determination of the sensitivity of the ^59^Co NMR peak to temperature (Δ*δ*/Δ*T*) was achieved *via* linear regression of the temperature-dependent data (see Fig. 5, S8, and Table S2†). These analyses revealed Δ*δ*/Δ*T* values for K_3_[Co(CN)_6_] and **1–5**, respectively, of 1.44(1), 1.44(2), 1.38(1), 1.30(2), 1.71(1), and 2.04(2) ppm °C^–1^. These values are within the ranges of sensitivity reported for the few ^59^Co NMR thermometers,^15^–^18^,^20^ but it's worth noting that, to the best of our knowledge, the Δ*δ*/Δ*T* of **5** is eclipsed only by Co(acac)_3_, a molecule that is completely unsuitable for aqueous (*e.g.* physiological) applications.^16^,^20^ Most importantly (and surprisingly), these data indicate that the highest sensitivity to changes in temperature is held by the completely encaged complex **5**.

The values of Δ*δ*/Δ*T* follow an opposing trend to the initial hypothesis, in that **5**, with the highest degree of encapsulation, displays the strongest Δ*δ*/Δ*T*. Complex **4**, with the second highest degree of encapsulation, displays the second highest sensitivity of our studied complexes. Yet, a comprehensive trend for all complexes on the basis of encapsulation is not indicated by these data. For example, in **1** and K_3_[Co(CN)_6_], the ligand donor atoms are not connected in any manner. Yet, these species demonstrate higher Δ*δ*/Δ*T* than both **2** and **3**, which contain bidentate chelates. Furthermore, the collected data show that electronic structure considerations alone (specifically, *Δ*_o_) do not govern sensitivity. Here, neither the trend in *Δ*_o_ extracted from UV-Vis (**3** < **5** < **1** < **4** < **2** < K_3_[Co(CN)_6_]) nor that from the 25 °C ^59^Co NMR (**3** < **1** < **4** < **2** < **5** < K_3_[Co(CN)_6_]) reproduce the trend in Δ*δ*/Δ*T* (see Fig. S9 and Table S3†).

The foregoing results highlight the need for deeper studies to derive fundamental insight. An important implication of the foregoing results is the concept that the encaged complex counterintuitively demonstrates the highest fluxionality in the inner-coordination structure. Four key experiments were applied to further test this rationale.

**Fig. 6:**
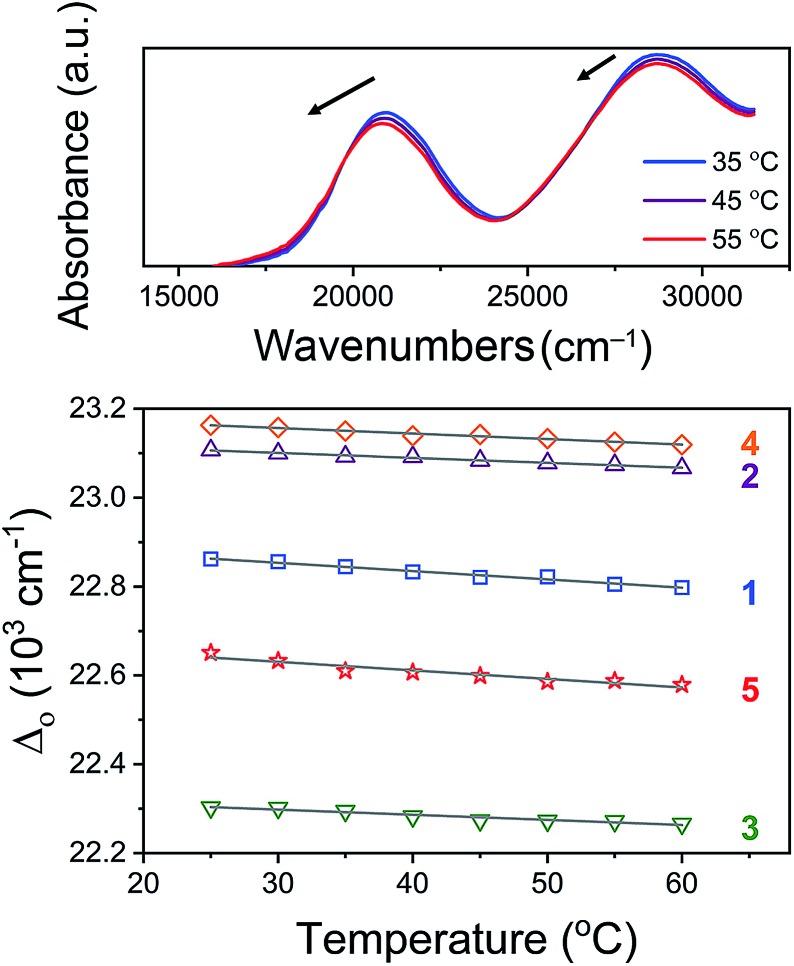
Variable temperature UV-Vis analysis. Top: normalized variable-temperature spectra for **5** in H_2_O at 4.5 mM concentration. Bottom: temperature dependence of *Δ*_o_ for **1–5**. Linear regression yielded the grey lines and Δ*Δ*_o_/Δ*T* – see main text for more detail.

If the molecular structure of [Co(diNOsar)]Cl_3_ is truly more temperature-dependent than **1–4**, then *Δ*_o_ for **5** should show the greatest temperature dependence. Variable-temperature UV-Vis spectra for **1–5** show slight shifts to lower energy with increasing temperature (Fig. 6 and S10–S12†). Analyses of these data reveal a change in *Δ*_o_ as a function of temperature, Δ*Δ*_o_/Δ*T*. Over **1–5**, Δ*Δ*_o_/Δ*T* assumes values of –2.78(4), –1.36(17), –2.91(5), –3.70(17), and –5.65(32) cm^–1^ °C^–1^ for **1–5** respectively. These spectral changes are consistent with studies probing temperature-dependent UV-Vis spectra for metal complexes wherein spin-state changes are absent^36^,^37^ (*versus* systems displaying spin-crossover^38^ or valence tautomerization^39^). These data trend in a manner (particularly for **2–5**) that seems opposed to an association between encapsulation and increased rigidity of the coordination environment. Indeed, **5** exhibits the largest change in temperature, followed by **4**, then **3** and **1**, and finally **2**. Hence, these data point to a more dynamic inner coordination sphere.

**Fig. 7:**
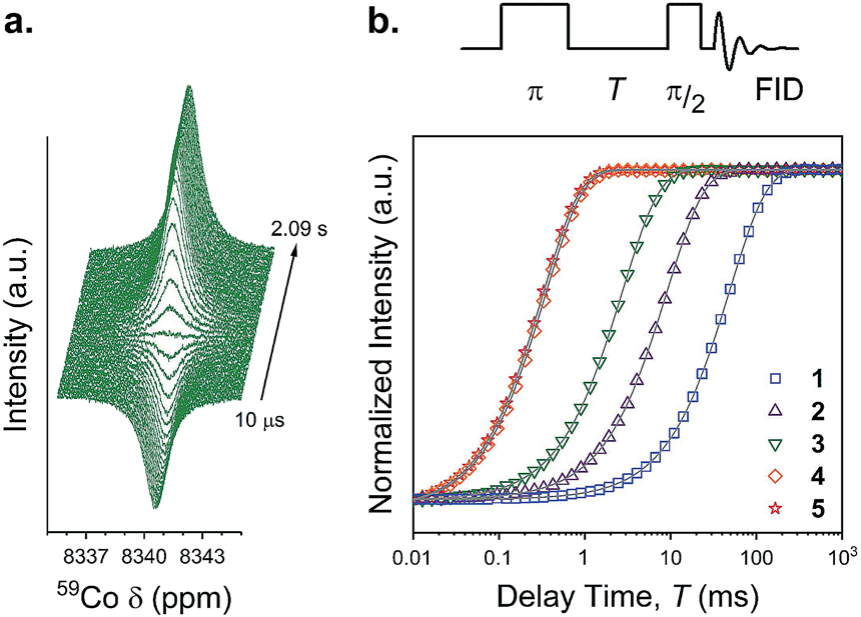
Spin-lattice relaxation data for **1–5**. (a) ^59^Co NMR spectrum as a function of delay time for **3** following the 22.5 μs inversion pulse. (b) Specific inversion recovery sequence used, and intensity of the peaks as a function of the delay time. Grey lines are exponential fits to the data, yielding *T*_1_. See main text for more information on the fits. All data were collected at 25 °C and 33 mM concentration.

If the inner coordination sphere is less rigid upon encapsulation, then cobalt-59 spin lattice relaxation times should reflect that point. Indeed, the *I* = 7/2 cobalt-59 nucleus is quadrupolar, and, hence, its spin-lattice relaxation rate (1/*T*_1_) is dominated by fluctuations in the local electric field gradient.^40^ Hence, the anticipated higher fluxionality in the CoN_6_ environment of **4** and **5***versus***1–3** should correspondingly engender shorter *T*_1_. Analysis of the inversion recovery traces for **1–5** (Fig. 7) reveal *T*_1_ parameters for **1–5** that follow a trend with encapsulation, wherein [Co(NH_3_)_6_]Cl_3_ (**1**) displays the longest *T*_1_ (48.47(5) ms), followed by **2** (9.09(2) ms) and **3** (2.73(1) ms). In contrast, the species of highest encapsulation, **4** and **5**, have the shortest *T*_1_ values (346(1) and 323(1) μs, respectively). For **1** and **2**, these values match previously reported results.^40^–^42^ Quadrupolar relaxation is also enhanced in systems with a higher ^59^Co quadrupole coupling constant, and this constant is smaller for high-symmetry complexes.^9^ Compound **1** is clearly higher symmetry (*O*_h_) than **2–5** (*D*_3_). This symmetry difference is likely an important contributor to the *T*_1_ of **1***versus***2–5**, but quadrupolar couplings in this latter set of compounds are similar (when known).^42^,^43^ Moreover, solution-phase rotational rates for the series extrema, **1** and **5**, are similar,^42^ suggesting rotational correlation is also not driving the difference in *T*_1_ across the series. Together, these points suggest that considerations beyond symmetry/rotation define *T*_1_ for these compounds. In light of the other data in this paper, we propose that the *T*_1_ trend evidences a more dynamic coordination environment upon encapsulation, though deeper investigations are needed to test this hypothesis.

**Fig. 8:**
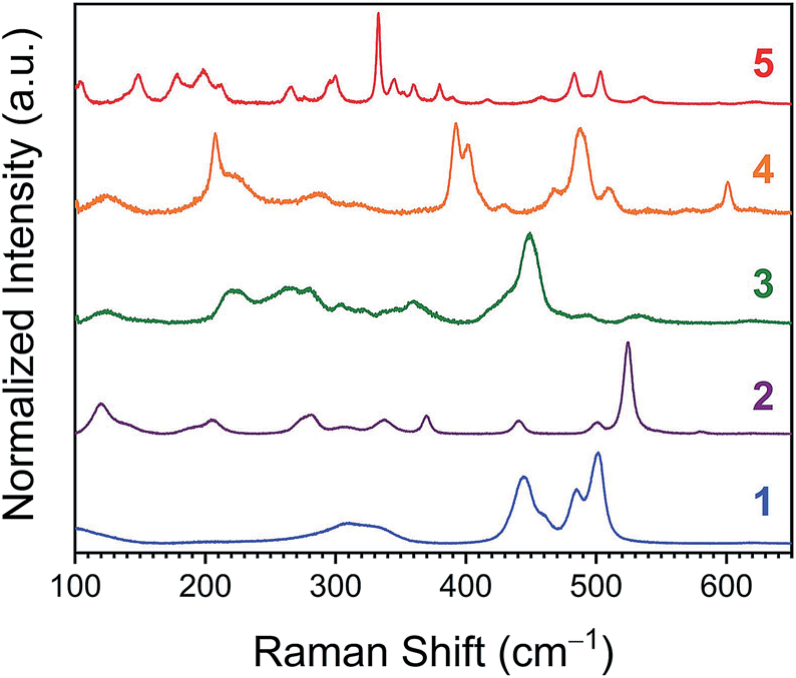
Room-temperature Raman spectra collected on pure powder samples of **1–5**. All spectra are baseline corrected.

Vibrational spectra in the 100–650 cm^–1^ window, wherein metal–ligand vibrations typically occur, ought to vary with rigidity as well.^44^ To test this concept, microcrystalline powders of **1–5** were analyzed *via* Raman spectroscopy. As the probed molecules increase in structural complexity, so do the Raman spectra, with compound **1** exhibiting 8, **2** displaying 12, and **5** producing 21 bands below 650 cm^–1^ (Fig. 8). Previous reports identify symmetric Co–N bond stretches at 500 and 486 cm^–1^ for **1**, and 526, 444, and 476 cm^–1^ for **2**.^45^,^46^ For **3–5**, in contrast, no Raman spectra are reported to the best of our knowledge. Closer inspection of the Raman spectra reveals a general sharpening of transitions with increasing encapsulation. This sharpening is most noticeable when comparing the spectra of lesser encapsulated compounds **1–3** with the completely encapsulated species **5**. Linewidth analyses of the observed vibrations for **1–5** permitted relative quantitation of the general degree of sharpness of these spectra (Fig. S13–S17 and Table S4†). The averages of the peak linewidths for the spectra are ordered from **5** < **2** < **4** < **1** < **3**, where the fully encapsulated species, **5**, exhibits the smallest average peak width.

On the basis of the variable-temperature NMR and UV-Vis data, the highest flexibility is observed for **5**. However, the lifetime of the NMR experiment is much longer than that of vibrational spectroscopy.^47^ Hence, one may therefore expect the greater structural variability from the NMR/UV-Vis analyses to result in greater inhomogeneous broadening of the vibrational peaks for **5**. The observations from the Raman spectra are in contrast to this expectation, as **5** demonstrates the sharpest peaks. One alternative mechanism that governs peak linewidths is homogeneous broadening, which causes sharper peaks for excitations that have longer lifetimes.^48^ This mechanism is acknowledged as dominant in studies of M(CO)_*n*_ at room temperature in solution.^49^,^50^ If operative and dominant in powders of **1–5**, this admittedly simplistic model of broadening would suggest that the lifetimes of the vibrations of the coordination sphere are enhanced by encapsulation. Translation of the average linewidths of **1–5** into average vibrational, spectroscopic lifetimes (*via* the relationship FWHM = 1/π*τ*) yields lifetimes of 0.4(2), 0.6(3), 0.4(2), 0.8(4), and 1.3(4) ps for **1–5**, respectively.

The foregoing linewidth interpretation should be treated with caution owing to three specific factors. First, differences in microcrystalline environment can have an important impact on Raman linewidths (*e.g.*ref. 51 and 52). We note, however, that a preliminary powder diffraction analysis of the same samples measured by Raman spectroscopy did not reveal a noticeable trend of crystallinity correlating to the observed lifetimes (Fig. S18†). Second, modes of differing symmetries can yield different linewidths,^49^ as is likely evidenced here in the spread of linewidths in the deconvoluted peaks. Third, true elucidation of the vibration lifetimes requires time-resolved methods, which would also help differentiate inhomogeneous *versus* homogeneous broadening mechanisms.^50^ These data clearly motivate further solution-phase, time-resolved vibrational studies, a critical component of planned follow up work. Nevertheless, the obtained lifetimes are in the general picosecond range expected for metal complexes.^49^,^50^

If encapsulation affects Δ*δ*/Δ*T via* modulating vibration lifetimes, that insight would provide a new design principle for vibrational control of molecular spin. Variable-solvent studies of **2** were performed as one final test of this concept. In particular, as the polar N–H bonds of the coordinated nitrogen atoms in **1–5** likely interact with the aqueous environment, this interaction should mediate the vibrations and structure of the [Co(en)_3_]^3+^ moiety, potentially imparting large differences to Δ*δ*/Δ*T*. Indeed, such hydrogen bonding interactions are demonstrated to enable modulation of M–N and M–O bonds in other molecular systems.^53^,^54^ Here, this concept is being tested for temperature-dependent magnetic effects.

**Fig. 9:**
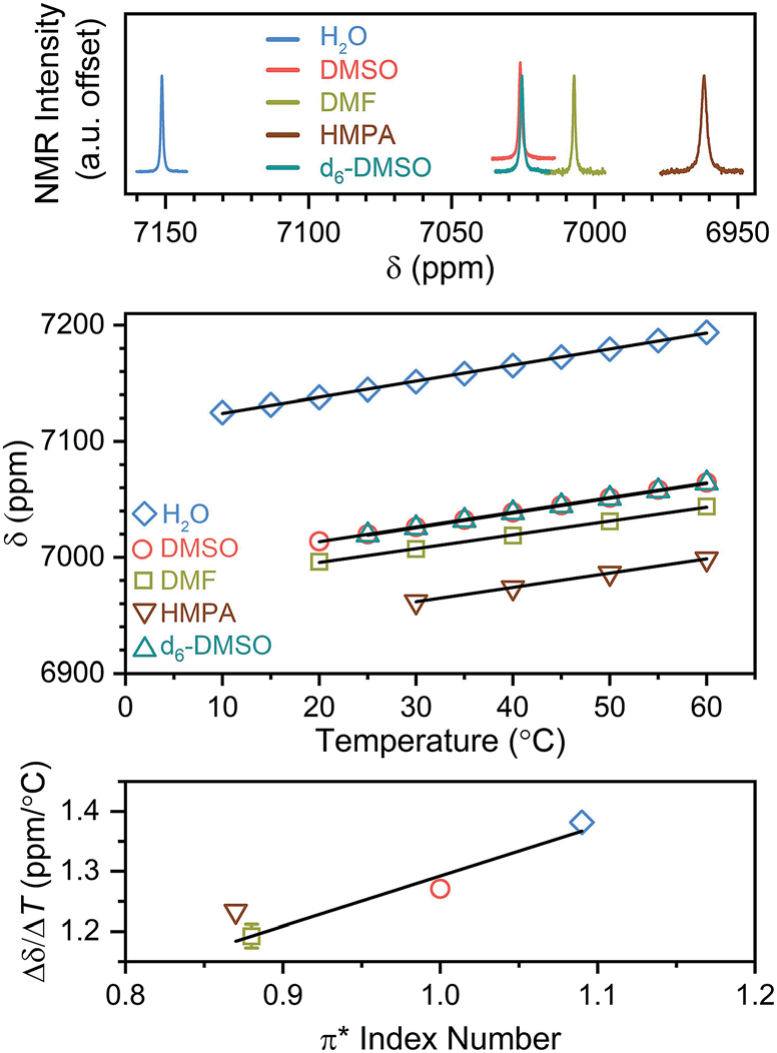
Variable-solvent ^59^Co NMR studies of [Co(en)_3_]Cl_3_ (**2**). Top: ^59^Co NMR spectra of **2** in solvents of differing polarity. All data are reported at 25 °C from a 500 MHz NMR. Middle: temperature dependence of peak position as a function of solvent for **2**. Solid lines are the result of linear regression to yield Δ*δ*/Δ*T* (see main text). Bottom: sensitivity of ^59^Co *δ* to changes in temperature as a function of solvent π* polarity index. Error bars are less than the symbol size, except for DMF. The solid line is the result of linear regression with *R*^2^ = 0.9.

Initial studies focused on one member of the series, [Co(en)_3_]Cl_3_ (**2**), dissolved in four additional solvents: dimethylformamide (DMF), hexamethylphosphoramide (HMPA),§
§HMPA is a Class 1B carcinogen and mutagen and should be handled with extreme care. dimethylsulfoxide (DMSO) and d_6_-dimethylsulfoxide (d_6_-DMSO). The solvents DMF, HMPA, and DMSO were selected to test polarity, and d_6_-DMSO chosen to test the impact of environmental deuteration. While solvent/deuteration impacts on ^59^Co *δ* are reported,^55^ their role on thermometry is not yet understood. ^59^Co NMR spectra collected at 25 °C reveal a peak position that shifts over a range of 200 ppm as a function of solvent identity (see Fig. 9). This solvent-dependent effect is known for the ClO_4_^–^ salt of the [Co(en)_3_]^3+^ cation, stemming from modulation of the N-atom ligand field *via* hydrogen bonding between the solvent and N–H protons.^56^ Furthermore, only a tiny shift in *δ* is observed between DMSO and d_6_-DMSO, also in line with expected results.^55^

Variable-temperature analyses tested the impact of these differing solvent cages on Δ*δ*/Δ*T* (Fig. S19–S22 and Table S5†). Here, analysis of the variable-temperature ^59^Co NMR peak positions as a function of solvent demonstrate a noticeable impact of solvent identity on Δ*δ*/Δ*T* (Fig. 9). As in H_2_O, all ^59^Co NMR chemical shifts move downfield with increasing temperature. Linear regression of these temperature-dependent data reveal Δ*δ*/Δ*T* values of 1.19(2), 1.23(1), 1.27(1), and 1.28(1) ppm °C^–1^, respectively, for DMF, HMPA, DMSO, and d_6_-DMSO. These values are all lower than in H_2_O (Δ*δ*/Δ*T* = 1.38(1) ppm °C^–1^) and indicate nearly no role for solvent deuteration on Δ*δ*/Δ*T* in the present compound.

Comparison of the solvent-dependent Δ*δ*/Δ*T* results for **2** against measures of solvent–solute interaction potentially provide deeper insight into the role of the solvent cage (Fig. 9, S23 and Table S5†). In particular, the trend in Δ*δ*/Δ*T* was contrasted against (1) the solvent acceptor and donor numbers,^57^,^58^ (2) the π* solvent polarity scale,^59^,^60^ and (3) the β and α hydrogen-bonding donor/acceptor scales.^60^–^62^ There may be an approximate correlation between Δ*δ*/Δ*T* and acceptor number, whereas there is clearly none for donor number and little, if any with β (see Fig. S23†). Analysis with *α* values is complicated as *α* is 0 for all solvents here except H_2_O.^60^ However, the π* scale clearly reveals a correlation (*R*^2^ = 0.9, Fig. 9, bottom).

The foregoing data point toward a coordination environment that is counterintuitively more flexible and dynamic with increasing encapsulation. Electron transfer studies of sarcophagine-like ligands provided the first assertions of rigidity in encapsulated Co(iii) complexes based on a conformationally inflexible environment.^63^–^67^ We propose that this conformation-based description of rigidity is insufficient for understanding the trend of Δ*δ*/Δ*T*. Instead, we tentatively posit an alternative, spin-relevant interpretation in this context. Here, the enhanced connectivity in **4** and **5** ensures a higher rigidity in the coordination environment, except in this case the rigidity permits vibrations of the encapsulated coordination environment to persist longer. Such longer lifetimes ultimately sustain a change in the coordination sphere by lengthening the equilibrium Co–N bond distances. Hence, there is a temperature dependence of *Δ*_o_ and *δ* in **4** and **5** that is larger than **1–3**. This tentative interpretation of the data is also consistent with the solvent dependence of Δ*δ*/Δ*T* in **2**, as the solvent cage is known for impacting vibration lifetimes in coordination complexes.^49^,^68^,^69^ The fundamental argument we propose here is an analogue to the justification of long phonon lifetimes in materials like diamond,^70^,^71^ except here related to the molecular vibrations of a complex in solution. These studies clearly motivate future investigations to evaluate the validity of this picture of vibration-controlled spin properties.

Furthermore, the solvent-dependent data hint at a rich area of inquiry into the role of the second coordination sphere and counterions. When considering the [Co(en)_3_]^3+^ unit, interactions with the solvent are most easily intuited *via* the N–H protons accepting electron density from solvent molecule lone pairs. The association of a higher Δ*δ*/Δ*T* with a higher π* index of solvent polarity^59^ would mesh with this intuited picture. This model would also be consistent with the match between a lower *β* value and a higher Δ*δ*/Δ*T*, as a low *β* occurs when a solute will only weakly accept a proton.^62^ Thus, these data suggest that the N–H interactions are key to understanding Δ*δ*/Δ*T*. However, the interpretation isn't without some uncertainty. To the extent that there is any correlation of Δ*δ*/Δ*T* with solvent properties, it is with their acceptor number, not donor number, meaning that [Co(en)_3_]^3+^ acts as a donor. This argument only makes sense if one also considers the lone pairs of a bound Cl^–^ counterion, not the N–H bonds. Indeed, earlier studies of [Co(en)_3_]^3+^ and [Co(diNOsar)]^3+^ demonstrate a close association between these species and their Cl^–^ counterions that persists in solution.^43^,^67^,^72^ Noted reservations about generalizing the acceptor/donor number scale lend caution to the second explanation of the solvent-dependent data.^73^ Nevertheless, the conflict between these two interpretations underlines the necessity of further investigations into the role of the counterion and solvent cage on Δ*δ*/Δ*T*.

## Conclusions and outlook

The foregoing results are the first evidence of synergy between ligand encapsulation and enhanced temperature-dependent magnetic changes in metal-ion nuclear spins. Such knowledge is of broad impact, as exploiting molecular rigidity to control magnetism is an emerging trend in designing molecules for other spin-based technologies, *e.g.* molecular quantum bit development.^74^–^76^ Importantly, the presented arguments potentially tie vibration lifetimes to nuclear magnetism – necessitating future time-resolved measurements to test the validity of this analysis. Finally, our studies reveal that in addition to the ligand, manipulations of the counterion and solvent cage are the next stage for understanding the mechanisms that control Δ*δ*/Δ*T*. Beyond the targeted applications in thermometry, the concepts herein could be extended to understanding the impacts of molecular rigidity on other spin-based applications, for example, designing electron paramagnetic resonance imaging probes,^77^ rigid systems for dynamic nuclear polarization^78^–^80^ (particularly with metal ions),^81^,^82^ or molecular quantum sensors.^83^,^84^

## Conflicts of interest

There are no conflicts to declare.

## Supplementary Material

Supplementary information
