# Supplementary material for: Influence of ligand encapsulation on cobalt-59 chemical-shift thermometry
Source: Chem Sci. 2019 Jun 5;10(27):6727–34. doi: 10.1039/c9sc01689a (PMC6625495; doi:10.1039/c9sc01689a)
Supplement: Supplementary file 1 [file SC-010-C9SC01689A-s001.pdf]

Electronic Supplementary Material (ESI) for Chemical Science.
This journal is © The Royal Society of Chemistry 2019

Electronic Supplementary Information for:

Influence of Ligand Encapsulation on Cobalt-59 Chemical-Shift Thermometry

Tyler M. Ozvat, Manuel E. Peña, and Joseph M. Zadrozny

Department of Chemistry, Colorado State University, Fort Collins, Colorado 80523

Chem. Sci.

Table of Contents

Experimental details. S3-S5
Table S1: Results of Tanabe-Sugano Analyses. S8
Table S2: Table of variable-temperature chemical-shift and UV-Vis data. S9
Table S3: Table of $\Delta\delta/\Delta T$ and electronic structure parameters. S10
Table S4: Results of linewidth analyses of Raman spectra. S11
Table S5: Tables for variable-solvent studies. S12
Figure S1: Lambda max (λ_{max}) versus chemical shift (δ) plot for **1-5** and $K_3[Co(CN)_6]$. S13
Figure S2: Variable-temperature ^{59}Co NMR spectra for **1** in H_2O. S14
Figure S3: Variable-temperature ^{59}Co NMR spectra for **2** in H_2O. S15
Figure S4: Variable-temperature ^{59}Co NMR spectra for **3** in H_2O. S16
Figure S5: Variable-temperature ^{59}Co NMR spectra for **5** in H_2O. S17
Figure S6: Variable-temperature ^{59}Co NMR spectra for $K_3[Co(CN)_6]$ in H_2O. S18
Figure S7: Variable-temperature ^{59}Co NMR Linewidths for **1**–**5**. S19
Figure S8: Variable-temperature δ values for **1-5** normalized to 10 °C S20
Figure S9: Comparison of optical data and $\Delta\delta/\Delta T$ for **1-5** and $K_3[Co(CN)_6]$. S21
Figure S10: Variable-temperature UV-Vis data for **1** and **2** in H_2O. S22
Figure S11: Variable-temperature UV-Vis data for **3**, **4**, and **5** in H_2O. S23
Figure S12: Variable-temperature Δ_o normalized to 25 °C. S24
Figure S13: Deconvolution of Raman spectrum for **1.** S25
Figure S14: Deconvolution of Raman spectrum for **2.** S26
Figure S15: Deconvolution of Raman spectrum for **3.** S27
Figure S16: Deconvolution of Raman spectrum for **4.** S28
Figure S17: Deconvolution of Raman spectrum for **5.** S29
Figure S18: Powder diffraction data for samples of **1-5** used in Raman analysis. S30
Figure S19: Variable temperature ^{59}Co NMR spectra for **2** in DMSO. S31
Figure S20: Variable temperature ^{59}Co NMR spectra for **2** in d_6-DMSO S32
Figure S21: Variable temperature ^{59}Co NMR spectra for **2** in DMF. S33
Figure S22: Variable temperature ^{59}Co NMR spectra for **2** in HMPA. S34
Figure S23: Comparison of $\Delta\delta/\Delta T$ for **2** with different solvent-solute interaction scales. S35
References S36

Experimental Section

General Considerations. Compounds were synthesized as previously reported unless otherwise specified. Compounds **1** and $K_3[Co(CN)_6]$ were purchased from commercial vendors and recrystallized prior to measurement. 1,1,1-Tris(aminomethyl)ethane trihydrochloride (tame•3HCl) was synthesized from a previously reported procedure.[1] All other precursors and chemicals were purchased from commercial vendors and used as received.

$[Co(en)_3]Cl_3$•$3H_2O$ (2•$3H_2O$**)** This complex was synthesized following a previously published procedure.[2] A crystalline, yellow-orange solid was obtained (7.071 g, 40.5% yield) after air drying. IR (cm^{-1}, diamond ATR): 438 (vs), 471 (m), 580 (w/s), 706 (s/vw), 780 (s), 898 (s/vw), 1057 (s), 1124 (s/w), 1156 (s), 1254 (s/vw), 1280 (s/vw), 1326 (s/w), 1364 (s/w), 1439 (m), 1463 (s), 1561 (s), 1583 (m), 3096 (s), 3204 (m), 3484 (m). UV-Vis (H_2O, Fig. 2) match reported data:[3] λ_{max} (nm) (ε_M ($M^{-1}cm^{-1}$)): 338 (81) and 465 (83). Anal. Calcd. (Found) for $C_6H_{24}Cl_3CoN_6$•$3H_2O$: 18.03 (17.59) %C, 7.57 (7.55) %H, and 21.02 (20.95) %N.

$[Co(pn)_3]Cl_3$•$3.5H_2O$ (3•$3.5H_2O$**)** This complex was synthesized using a modified literature procedure.[4] Here, $CoCl_2$•$6H_2O$ (6.0 g, 0.025 mol) and 8.5 mL of 85% 1,3-propylenediamine (pn) were dissolved in 20.0 mL of water. To this solution, 2.10 mL of conc. HCl were added slowly followed by 0.5 g of activated charcoal. The solution was aerated with stirring for 18 hr, then filtered and the collected solid was washed with 3 × 7 mL of H_2O. To the filtrate and washings were added 95 mL of MeOH and 50 mL of Et_2O. The precipitate crashed out of solution and was filtered to yield a crystalline, salmon-colored solid (9.80 g, 28.0%). Collected spectra match reported data.[5] IR (cm^{-1}, diamond ATR): 420 (s), 448 (w/s), 488 (w/s), 515 (w/s), 520 (s), 690 (m/w), 720 (s/vw), 733 (s/vw), 886 (s/w), 931 (s), 1039 (vs), 1088 (s/vw), 1138 (m/vw), 1185 (s), 1208 (s/w), 1234 (s), 1274 (m/w), 1310 (s/vw), 1362 (m/w), 1413 (m/w), 1456 (s/vw), 1478 (w/s), 1571 (m), 2888 (w/s), 2962 (m), 3090 (s), 3151 (m), 3392 (m/w). UV-Vis (H_2O, Fig. 2): λ_{max} (nm) (ε_M ($M^{-1}cm^{-1}$)): 350 (76) and 485 (74). Anal. Calcd. (Found) for $C_9H_{30}Cl_3CoN_6$•$3.5H_2O$: 23.98 (24.11) %C, 8.27 (8.06) %H, and 18.64 (18.59) %N.

$[Co(tame)_2]Cl_3$•$1.5H_2O$ (4•$1.5H_2O$**)** The trihydrate was synthesized using a modified literature

procedure.[5] tame•3HCl (0.645 g, 2.85 mmol) was dissolved in 5 mL of water and mixed with Ag_2O (1.33 g, 5.74 mmol). The mixture was covered in aluminum foil and stirred for 2 hr. The resulting mixture was filtered to obtain a colorless solution, which was then added to a mixture of $CoCl_2$•$6H_2O$ (0.239 g, 1.00 mmol) and activated charcoal (0.043 g). After 1 min, 0.5 mL of conc. HCl were added and the solution was stirred for two hours under gentle aeration. The resulting orange-brown solution was then filtered through a pad of celite into dilute HCl (2.5 mL, 1 M). This solution was concentrated, filtered, and placed in an ice bath, forming bright orange, hexagonal crystals. The crystals were filtered and washed with ~15 mL of acetone and ethanol to obtain a bright orange, crystalline solid (0.355 g, 46.2%). Collected spectra match reported data.[3] IR (cm^{-1}, diamond ATR): 424 (vs), 571 (vw/s), 745 (m/vw), 815 (m/vw), 888 (m/vw), 908 (s/w), 1009 (s), 1068 (s/vw), 1128 (s/vw), 1153 (s/w), 1227 (m), 1310 (m/vw), 1349 (s/vw), 1373 (m/vw), 1401 (s/vw), 1460 (s/w), 1500 (m/vw), 1545 (m/vw), 1594 (m), 1600 (m), 2884 (s/w), 2947 (m), 3027 (s), 3150 (m), 3499 (m). UV-Vis (H_2O, Fig. 2): λ_{max} (nm) (ε_M ($M^{-1}cm^{-1}$)): 338 (67) and 468 (78). Anal. Calcd. (Found) for $C_{10}H_{30}Cl_3CoN_6$•$1.5H_2O$: 28.15 (28.31) %C, 7.79 (7.45) %H, and 19.64 (19.62) %N.

[Co(sarc)]Cl_3•$2H_2O$ (5•$2H_2O$**)** This complex was synthesized following a previously published procedure.[6] The resultant orange, powdery compound (4.300 g, 55.0%) revealed spectra that match reported data.[7] IR (cm^{-1}, diamond ATR): 438 (s/w), 467 (s), 503 (m), 597 (vs/w), 621 (s/vw), 787 (vs/w), 812 (vs), 840 (s/w), 873 (s/w), 952 (s/w), 974 (m/vw), 1019 (s/w), 1060 (m), 1077 (m), 1131 (m/vw), 1171 (s/vw), 1198 (s/vw), 1236 (s/vw), 1271 (s/vw), 1342 (s), 1382 (s/vw), 1429 (s/vw), 1453 (m), 1555 (vs), 1602 (m/w), 1655 (m/vw), 2375 (m/vw), 2947 (m), 3004 (m/w), 3032 (m), 3409 (m/w), 3466 (m/w). UV-Vis (H_2O, Fig. 2): λ_{max} (nm) (ε_M ($M^{-1}cm^{-1}$)): 342 (204) and 475 (161). Anal. Calcd. (Found) for $C_{14}H_{30}Cl_3CoN_8O_4$•$2H_2O$: 29.21 (29.32) %C, 5.95 (5.70) %H, and 19.46 (19.36) %N.

NMR Measurements. NMR spectra were obtained at Colorado State University (CSU) on a Varian UnityINOVA 500. All variable temperature NMR measurements for $K_3[Co(CN)_6]$ and compounds **1-4** were collected in 100 mM solutions of H_2O, with the exception of compound **5**, which was measured in a 33.3 mM solution. Experiments performed as a function of concentration reveal no difference in $\Delta\delta / \Delta T$ for **1-5** in this concentration range. For variable solvent studies, the

NMR measurements were acquired for compound **2** in ~20 mM solutions of DMSO and d_6-DMSO, ~15 mM solution of DMF, and ~10 mM solution of HMPA (**CAUTION:** HMPA is a Class 1B carcinogen and mutagen and should be handled with extreme care). These concentrations were selected on the basis of highest level of solubility for **2** in these solvents. For all measurements, standards of methanol and ethylene glycol were used to monitor and accurately determine the temperature inside of the magnet.[6] Samples were permitted to equilibrate for 5-10 minutes prior to analysis following insertion or temperature changes. Data were processed using Mestrenova[8] and OriginPro.[9] Owing to the low concentrations/relatively high noise in the NMR spectra for **5** in H_2O and **2** in DMF and HMPA, a denoising filter (Non-local means)[10,11] was applied through Mestrenova to enhance the spectral resolution.

Spin-lattice relaxation times were collected via inversion recovery measurements. Samples of **1**–**5** were prepared at 0.33 mM concentration in H_2O. Samples were prepared on the bench and not deoxygenated, as such treatment has been shown to not have substantial effect on spin-lattice relaxation for the ^{59}Co nucleus.[12] Moreover, many other studies also omit the step,[13–16] putting our analyses on better footing for comparative study. Pulse lengths for the π and $^{\pi}/_{2}$ pulses were 22.5 and 11.25 μs, respectively, with a recycle delay generally 5 × the T_1 for the given system. Inversion recovery curves were successfully fit to a monoexponential function with OriginPro.

Variable-Temperature UV-Vis Spectroscopic Measurements. Compounds **1** – **5** were prepared as samples in water to concentrations of 9.5 mM (**1**), 6.7 mM (**2**), 5.9 mM (**3**), 6.5 mM (**4**), and 4.5 mM (**5**). Absorbance measurements were made with a DH-2000-BAL Ocean Optics UV/VIS NIR spectrometer equipped with T300-RT-UV/VIS Transmission dip probe of 1-inch path length. Temperature control of each sample was performed with a heated oil-bath monitored via thermocouple. Sample temperatures were monitored separately and allowed to equilibrate to the surrounding oil-bath. Upon equilibration, absorbance spectra for each sample were collected in intervals of 5 °C between 25 – 60 °C. For a given sample, the individual absorbance spectra were normalized and fit with a consistent baseline correction and Gaussian distribution to determine changes in peak maxima over temperature. Two absorption energies corresponding to $^1A_{1g} \rightarrow {}^1T_{1g}$ and $^1A_{1g} \rightarrow {}^1T_{2g}$ were present in each sample measurement. Absorption energies (E) were used to determine E/B (B = Racah parameter) on a d^6 Tanabe-Sugano diagram for all Co^{3+} compounds.

Using the lower energy transition $^1A_{1g} \rightarrow {}^1T_{1g}$, values of B were determined for each compound at each temperature interval, then used to calculate Δ_o, respectively (**Table S1**).

We note the small variation in concentration for these measurements. However, studies elsewhere demonstrate that the addition of salt doesn't modify the bulk properties of water.[17,18] Hence, the small variation in concentration shouldn't be significant and we can consider the molecules as being investigated under essentially the same conditions. Hence, the trend in $\Delta\Delta_o/\Delta T$ is truly dependent on the molecule itself. Preliminary variable-concentration studies, already mentioned with respect to $\Delta\delta/\Delta T$, also demonstrate that concentration changes over the range studied do not influence the variable-temperature spectroscopic properties here.

Raman Spectroscopic Measurements. Raman spectra of compounds **1** - **5** were collected at the CU Microspectroscopy Lab. Spectra were obtained using a Horiba LabRAM HR Evolution Spectrometer equipped with a 785 nm NIR laser (Nd:YAG). Each of the dried compounds were measured as powder samples individually loaded onto glass slides. All spectra were collected between 100 – 650 cm^{-1} by the same spectral resolution utilizing 1800 gr/mm grating and 24 mW laser power. Spectral deconvolution was performed in the Horiba LabSpec6 program on baseline-corrected spectra. Peaks were modeled with the pseudo-Voigt function (1), where A is peak amplitude, ω is peak width, x_0 is peak center, and g is the Gaussian linear mixing coefficient. Note that the character of the peak can be an indication of the mechanism of broadening: Gaussian for inhomogeneous broadening and Lorentzian for homogeneous broadening.[19,20] On the basis of the g values obtained from spectral deconvolution via eq (1) (Table S2), there is a distribution of different peak types in the observed spectra. These all point to the necessity of subsequent, deeper investigations to understand linewidth as a function of ligand identity.

$$V = A\left(g \cdot \exp\left(\frac{(x-x_0)^2}{\left(\omega/2\sqrt{ln\,(2)}\right)^2}\right) + (1-g)\cdot\frac{1}{1+(x-x_0)^2/(\omega/2\,)^2}\right) \quad (1)$$

All Other Characterization. General UV-Vis spectra were collected on aqueous solutions of **1**–**5** and $K_3[Co(CN)_6]$ with an Agilent 8453 UV-Visible spectrophotometer. IR spectra were collected on solid powders with a Bruker TENSOR II FTIR spectrometer. Combustion analyses were performed by Robertson Microlit Laboratories.[21–23,24] X-ray powder diffraction data were collected on powders of **1-5** loaded onto (510) cut zero-diffraction silicon wafers supported with grease.

Diffraction data were collected on a Bruker D8 Discover DaVinci Powder X-ray Diffractometer, operating in Bragg-Brentano geometry, using Cu K-alpha radiation, and a Lynxeye XE-T position-sensitive detector. Each compound was measured over a 2θ range from 5 and 70 degrees and 1 scan per sample.

Table S1 | Experimental values of B and Δ_o for compounds **1 – 5** over a temperature range of 25 – 60 °C. Values were calculated by a d^6 Tanabe-Sugano diagram using the maximum absorption energies collected from UV/Vis spectroscopy measurements.

	**1**		**2**		**3**		**4**		**5**	
T (°C)	B (cm^{-1})	Δ_o (10^3 cm^{-1})	B (cm^{-1})	Δ_o (10^3 cm^{-1})	B (cm^{-1})	Δ_o (10^3 cm^{-1})	B (cm^{-1})	Δ_o (10^3 cm^{-1})	B (cm^{-1})	Δ_o (10^3 cm^{-1})
25	631.09	22.86	596.45	23.11	593.23	22.27	609.96	23.12	578.02	22.65
30	631.92	22.86	596.38	23.10	592.37	22.27	610.26	23.11	576.37	22.63
35	631.67	22.85	598.20	23.09	594.96	22.26	607.19	23.10	579.92	22.61
40	632.97	22.83	597.57	23.09	595.42	22.26	612.07	23.11	582.28	22.61
45	633.83	22.82	596.99	23.08	596.79	22.25	608.56	23.11	580.44	22.60
50	634.15	22.82	598.97	23.08	599.16	22.23	610.32	23.09	583.21	22.59
55	635.47	22.80	602.01	23.07	596.97	22.24	612.90	23.09	585.29	22.59
60	635.71	22.80	598.71	23.07	598.40	22.24	613.99	23.09	584.78	22.58

Table S2 | Variable-temperature chemical shifts and UV-Vis peak positions.[a]

	1			2			3		
**T (°C)**	**^{59}Co δ(ppm)**	**ν_1 (10^3 cm^{-1})**	**ν_2 (10^3 cm^{-1})**	**^{59}Co δ(ppm)**	**ν_1 (10^3 cm^{-1})**	**ν_2 (10^3 cm^{-1})**	**^{59}Co δ(ppm)**	**ν_1 (10^3 cm^{-1})**	**ν_2 (10^3 cm^{-1})**
10	8222	-	-	7125	-	-	8318	-	-
15	8157	-	-	7131	-	-	8328	-	-
20	8164	-	-	7138	-	-	8334	-	-
25	8171	20.97	29.13	7145	21.40	29.29	8340	20.47	28.24
30	8178	20.94	29.11	7151	21.39	29.33	8346	20.45	28.25
35	8185	20.93	29.08	7158	21.35	29.28	8352	20.44	28.24
40	8192	20.92	29.08	7165	21.36	29.31	8358	20.41	28.25
45	8200	20.91	29.07	7173	21.35	29.32	8365	20.40	28.27
50	8207	20.92	29.06	7179	21.34	29.28	8372	20.38	28.24
55	8215	20.89	29.05	7187	21.33	29.29	8379	20.35	28.21
60	8222	20.87	29.03	7194	21.35	29.29	8386	20.34	28.22

	4			5		
**T (°C)**	**^{59}Co δ(ppm)**	**ν_1 (10^3 cm^{-1})**	**ν_2 (10^3 cm^{-1})**	**^{59}Co δ(ppm)**	**ν_1 (10^3 cm^{-1})**	**ν_2 (10^3 cm^{-1})**
10	7384	-	-	6864	-	-
15	7393	-	-	6872	-	-
20	7401	-	-	6883	-	-
25	7409	21.29	29.24	6894	20.93	28.83
30	7418	21.30	29.26	6904	20.91	28.78
35	7427	21.27	29.22	6914	20.92	28.76
40	7435	21.26	29.21	6924	20.89	28.74
45	7443	21.23	29.22	6934	20.89	28.73
50	7452	21.20	29.21	6944	20.85	28.74
55	7461	21.17	29.19	6953	20.83	28.70
60	7470	21.16	29.21	6963	20.81	28.67

[a]ν_1 and ν_2 represent the lowest and second-lowest observed transitions in the UV-Vis spectrum, respectively

Table S3 | Data for comparison of temperature sensitivity of the chemical shift versus ^{59}Co chemical shift.[a]

Compound	$\Delta\delta/\Delta T$ (ppm/°C)	^{59}Co δ (ppm)	$\lambda_{max(1)}$ (nm)	$\lambda_{max(2)}$ (nm)	ν_1 (10^3 cm^{-1})	ν_2 (10^3 cm^{-1})
$K_3[Co(CN)_6]$	1.44(1)	0	312	-	32.05	-
**1**	1.44(2)	8171	477	343	20.97	29.13
**2**	1.38(1)	7145	467	341	21.40	29.29
**3**	1.30(2)	8340	489	354	20.47	28.24
**4**	1.71(1)	7409	470	342	21.29	29.24
**5**	2.04(2)	6894	478	347	20.93	28.83

[a]Spectral data and ^{59}Co chemical shifts are reported for 25 °C

Table S4 | Summary of Raman linewidth data (ν, peak position, and Γ, linewidth, both in cm^{-1}, τ, lifetime = 1/πΓ, in picosecond units, *g* unitless) from deconvolution.[a,b,c]

Compound **1**				Compound **2**				Compound **3**				Compound **4**				Compound **5**			
ν	Γ	τ	g	ν	Γ	τ	g	ν	Γ	τ	g	ν	Γ	τ	g	ν	Γ	τ	g
105	33	0.32	0.00	119	20	0.53	0.76	124	25	0.43	0.00	124	41	0.26	1.00	104	12	0.87	0.89
278	52	0.21	0.00	136	33	0.32	1.00	221	31	0.35	1.00	208	8	1.35	0.58	139	10	1.11	0.17
306	42	0.25	1.00	189	22	0.47	1.00	259	70	0.15	0.86	219	53	0.20	0.85	148	11	1.01	0.53
331	45	0.23	1.00	205	20	0.54	0.70	265	21	0.51	0.97	284	56	0.19	0.66	178	15	0.70	0.72
444	25	0.42	0.75	278	26	0.40	0.88	282	20	0.53	0.89	319	22	0.49	1.00	198	21	0.50	0.54
461	15	0.70	1.00	307	26	0.40	1.00	303	10	1.04	1.00	392	10	1.08	0.76	212	9	1.18	1.00
484	17	0.64	0.38	336	21	0.50	0.48	314	49	0.22	1.00	402	10	1.02	0.00	265	10	1.04	0.88
501	16	0.66	0.71	369	9	1.12	0.64	341	18	0.60	1.00	429	9	1.13	1.00	276	7	1.58	0.00
				440	13	0.80	0.83	361	34	0.31	0.75	469	20	0.54	0.83	295	7	1.57	0.00
				500	13	0.82	1.00	435	46	0.23	1.00	488	19	0.55	0.90	300	6	1.92	0.00
				524	9	1.18	0.27	450	20	0.53	1.00	510	15	0.70	0.75	333	7	1.53	0.80
				580	10	1.09	1.00	462	53	0.20	1.00	601	7	1.63	0.00	345	8	1.30	0.34
								493	22	0.49	1.00					360	6	1.67	0.00
								533	27	0.40	0.56					380	5	2.12	0.01
								620	31	0.34	0.89					390	7	1.45	0.75
																416	9	1.16	0.97
																458	11	0.96	0.09
																483	7	1.44	0.00
																503	6	1.75	0.00
																536	11	0.96	0.11
																622	22	0.49	0.82

Table S5 | Data for comparison of temperature sensitivity of ^{59}Co δ for $[Co(en)_3]Cl_3$ in variable solvents.

	^{59}Co δ (ppm) of **2**				
T (°C)	H_2O	DMSO	d_6-DMSO	DMF	HMPA
20	7138	7014	-	6996	-
25	7145	7020	7019	-	-
30	7151	7026	7026	7007	6962
35	7158	7032	7032	-	-
40	7165	7039	7038	7019	6974
45	7173	7045	7045	-	-
50	7179	7051	7051	7031	6986
55	7187	7058	7057	-	-
60	7194	7065	7064	7044	6999

Solvent	Δδ/ΔT (ppm/°C)	π*[a]	AN[a]	DN[a]	β[a]
H_2O	1.38(1)	1.09	54.8	18	0.18
DMSO	1.27(1)	1.00	19.3	29.8	0.76
DMF	1.19(2)	0.88	16	26.6	0.69
HMPA	1.23(1)	0.87	10.6	38.8	1.05

[a]Values for solvent-descriptive parameters taken from refs 24 and 25.

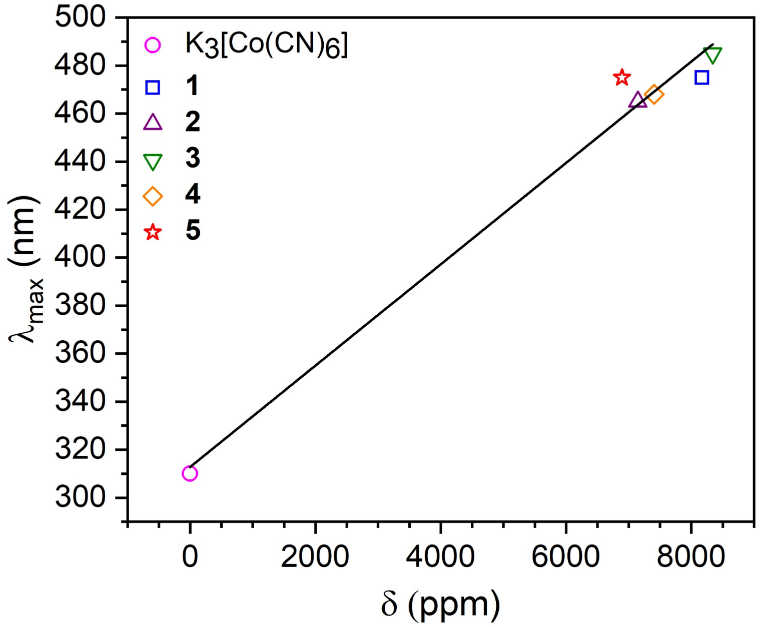

Figure S1 | Correlation between maximum wavelength of absorption (λ_{max}) and chemical shift (δ) for $K_3[Co(CN)_6]$ and compounds **1-5**. UV-Vis data were collected in water at a concentration of 10 mM for each sample. NMR data were collected in water at a concentration of 100 mM.

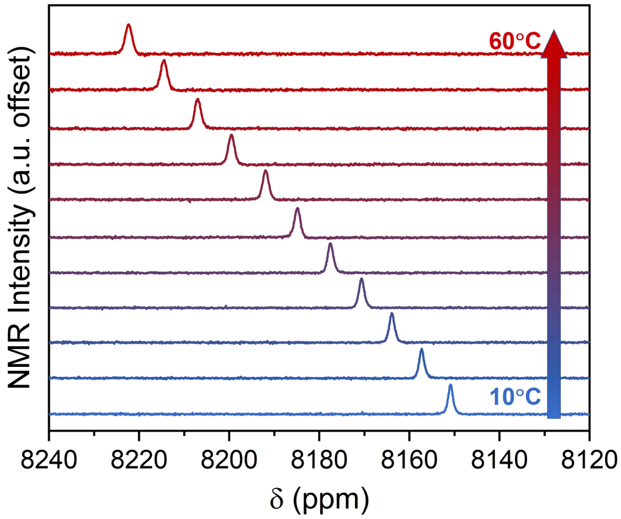

Figure S2 | Chemical shift data of compound **1** from 10-60 °C. NMR measurements were taken in a solution of H_2O at a concentration of 100 mM.

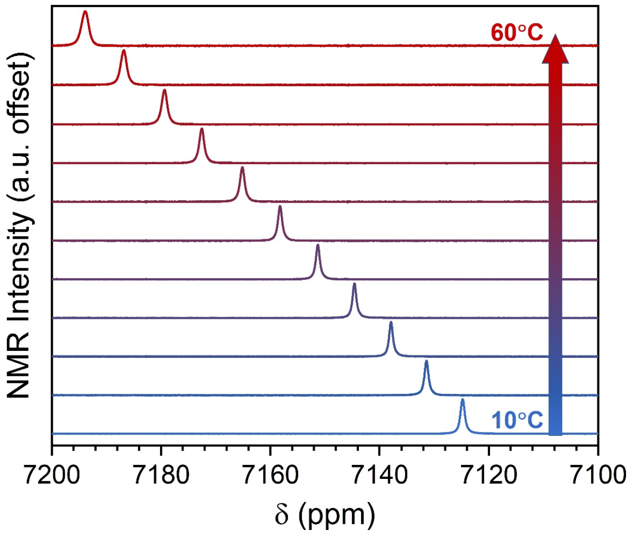

Figure S3 | Chemical shift data of compound **2** from 10-60 °C. NMR measurements were taken in a solution of H_2O at a concentration of 100 mM.

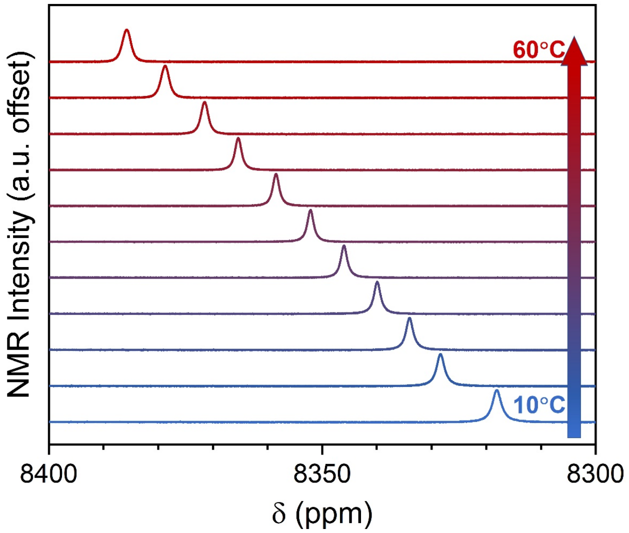

Figure S4 | Chemical shift data of compound **3** from 10-60 °C. NMR measurements were taken in a solution of H_2O at a concentration of 100 mM.

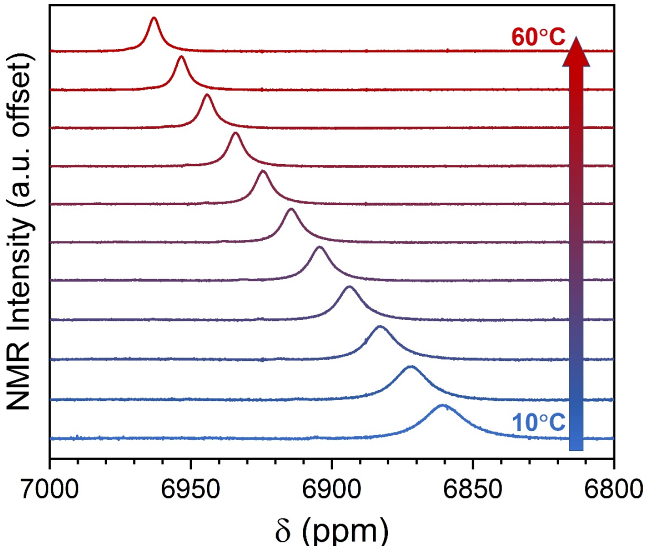

Figure S5 | Chemical shift data of compound **5** from 10-60 °C. NMR measurements were taken in a solution of H_2O at a concentration of 33.3 mM.

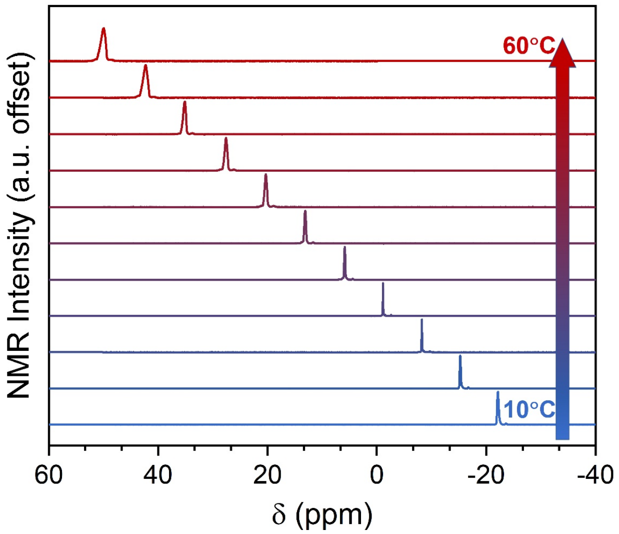

Figure S6 | Chemical shift data of $K_3[Co(CN)_6]$ from 10-60 °C. NMR measurements were taken in a solution of H_2O at a concentration of 100 mM.

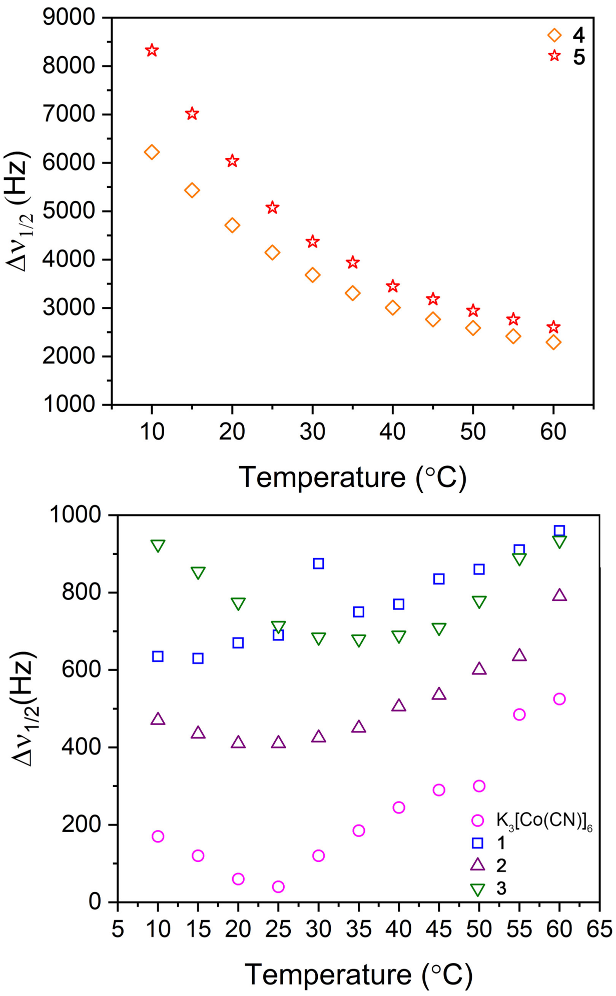

Figure S7 | Temperature dependence of the Co-59 NMR linewidths for **1**–**5**. Data were extracted from the full-width half maxima of the located peaks in the spectra. The linewidths here are quite large compared to other studies,[17-19] but these spectra were collected at higher field, and the results follow the expected field dependence.[18]

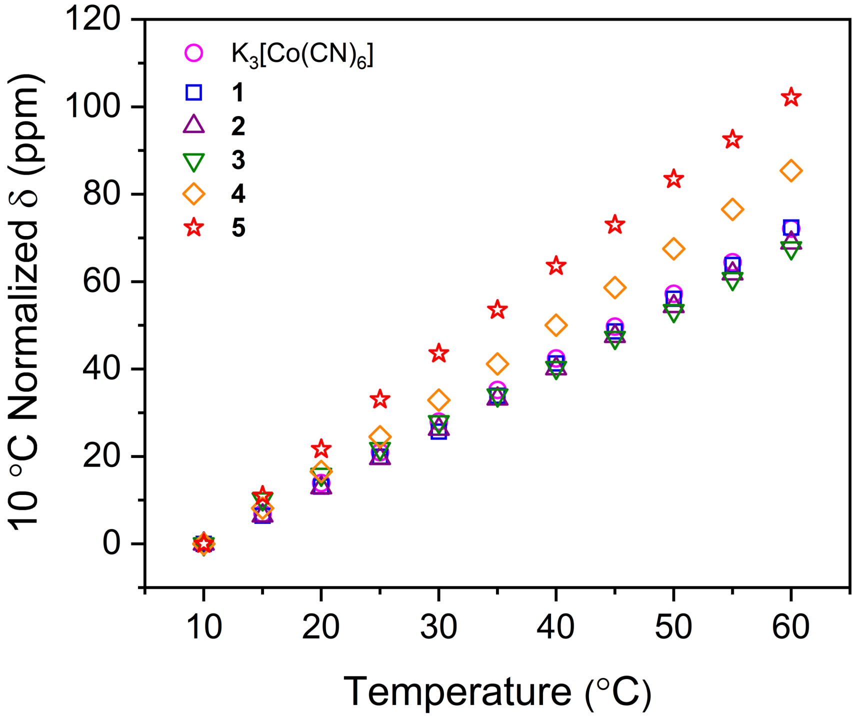

Figure S8 | Variable-temperature chemical shift data from 10-60 °C, normalized to 10 °C. NMR measurements were taken in a solution of H_2O at a concentration of 100 mM. Un-normalized data and linear regression are depicted in Fig. 5 of the main manuscript.

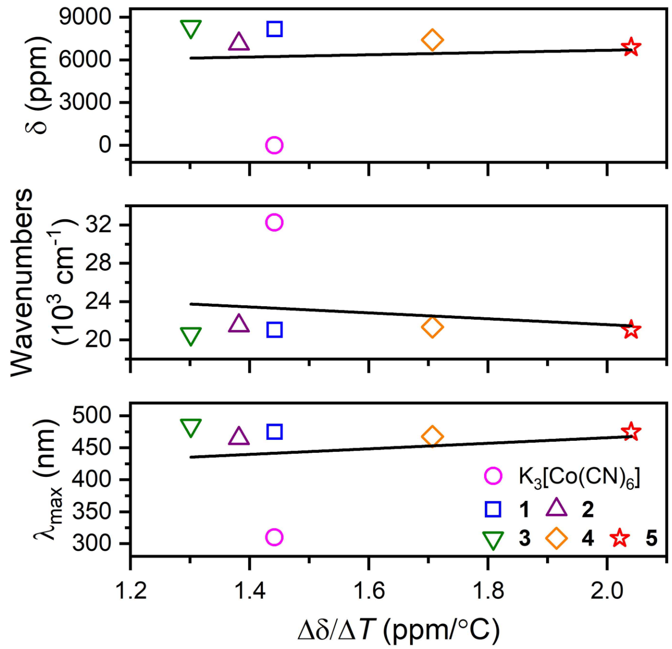

Figure S9 | Plots of chemical shift (δ, top) and UV-Vis data (in cm^{-1}, middle, and λ_{max}, bottom) as a function of $\Delta\delta/\Delta T$. UV-Vis data were collected in H_2O at a concentration of 10 mM for each sample. NMR data were collected in water at a concentration of 100 mM.

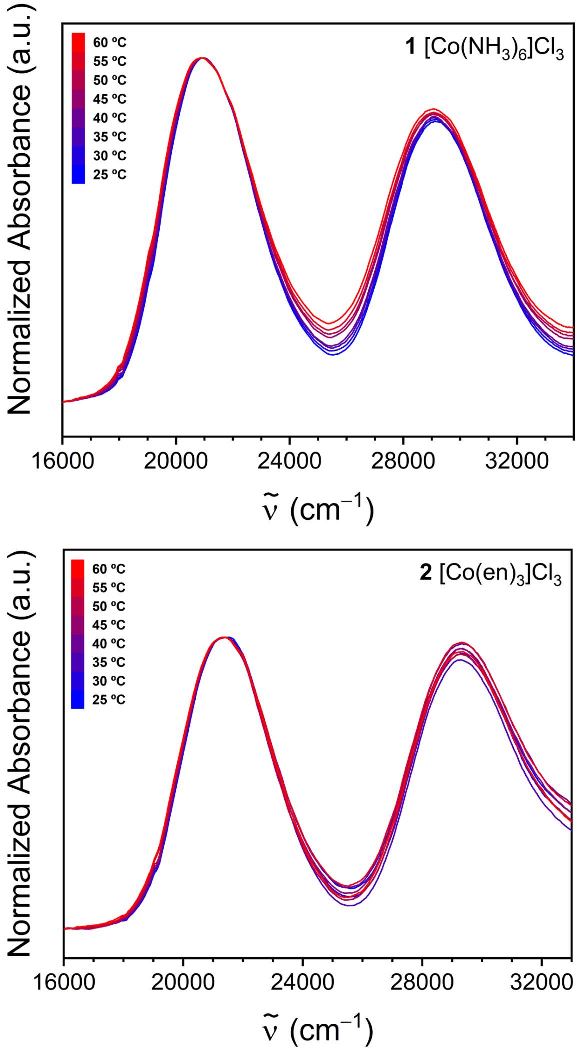

Figure S10 | Variable-temperature UV-Vis data for **1** and **2** in H_2O. Spectra were collected at concentrations of 9.5 mM (**1**) and 6.7 mM (**2**). Intensities were normalized for ease of view.

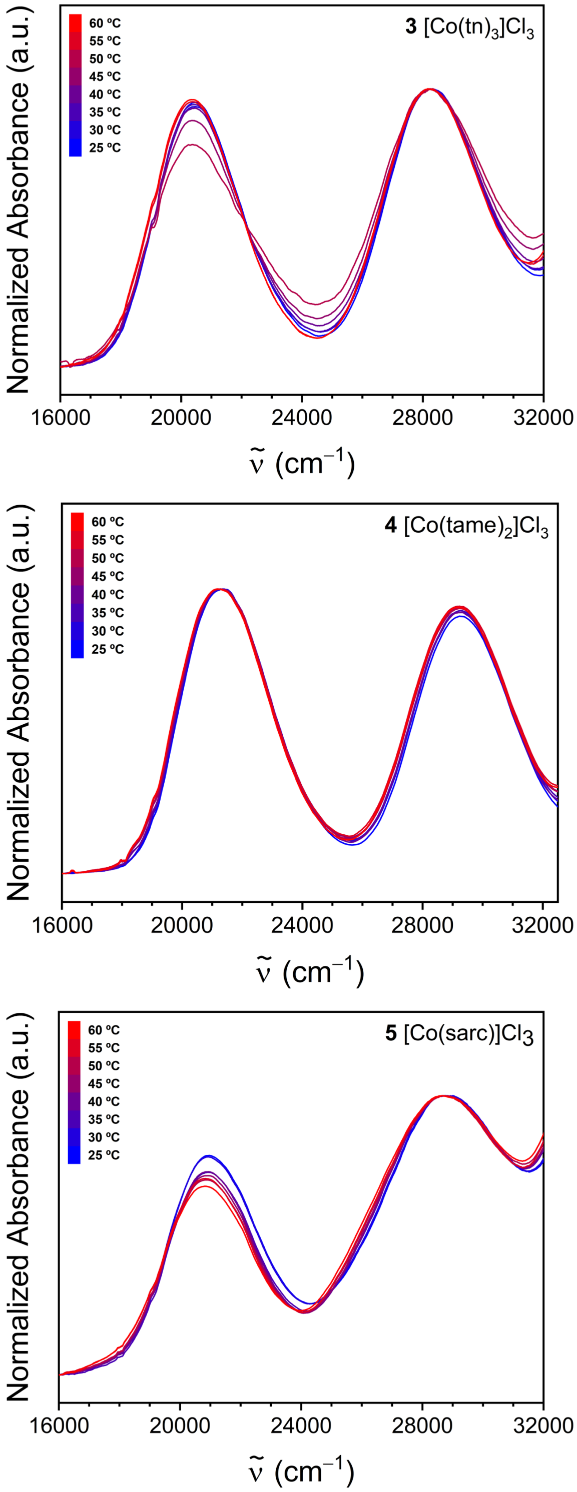

Figure S11 | Variable-temperature UV-Vis data for **3**, **4**, and **5** in H_2O. Spectra were collected at concentrations of 5.9 mM (**3**), 6.5 mM (**4**), and 4.5 mM (**5**). Intensities were normalized for ease of view.

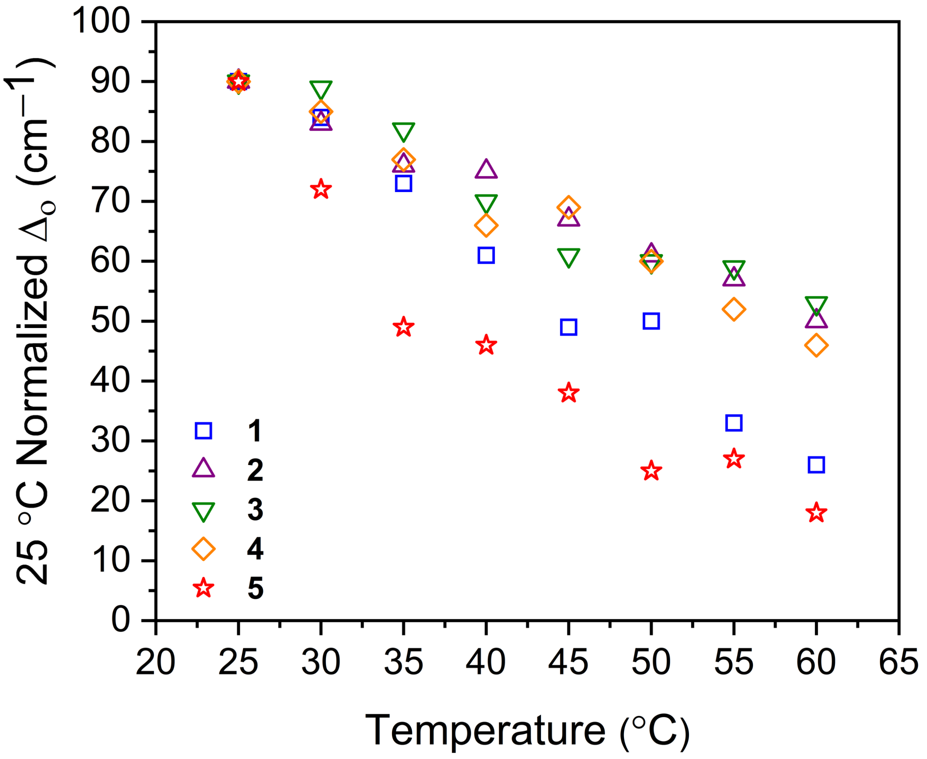

Figure S12 | Variable-temperature Δ_o normalized to 25 °C. Un-normalized data and regression analyses are reported in Fig. 6 of the main paper.

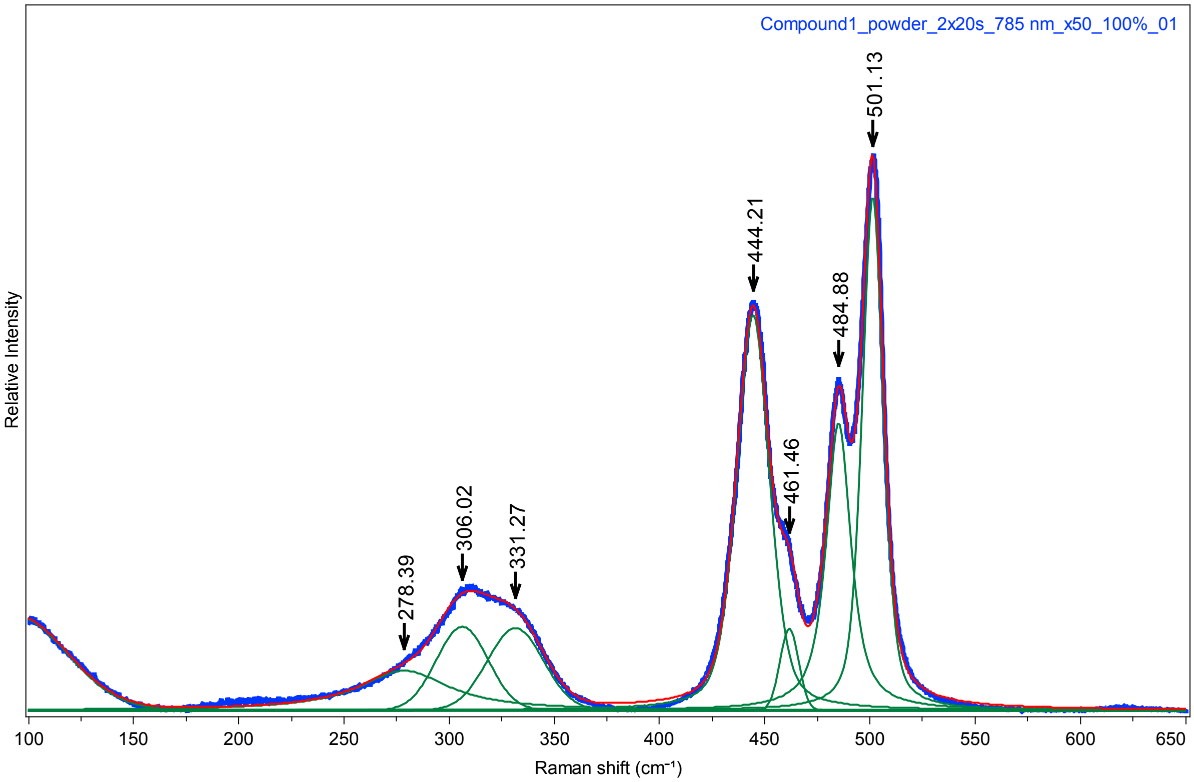

Figure S13 | Raman spectrum for a pure powder of **1** at room temperature. The data are presented in blue, the deconvoluted peaks are in green and the sum of the determined peaks is red, which overlays the experimental data.

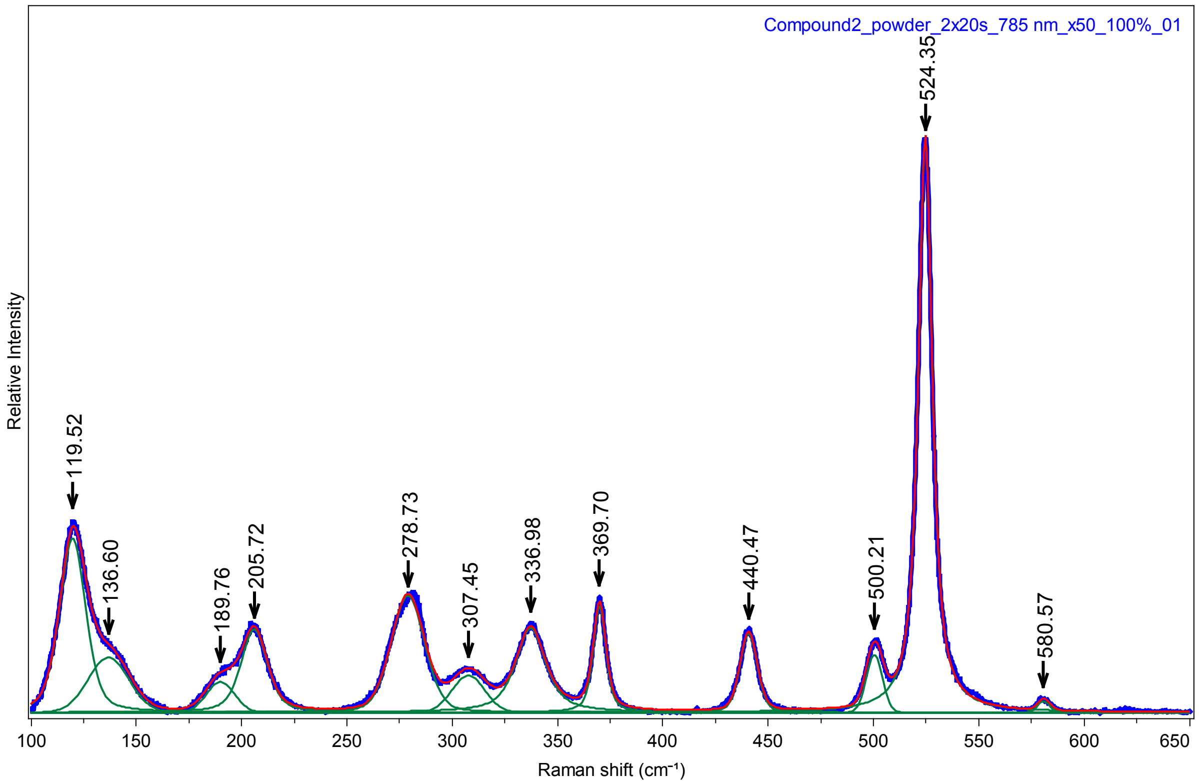

Figure S14 | Raman spectrum for a pure powder of **2** at room temperature. The data are presented in blue, the deconvoluted peaks are in green and the sum of the determined peaks is red, which overlays the experimental data.

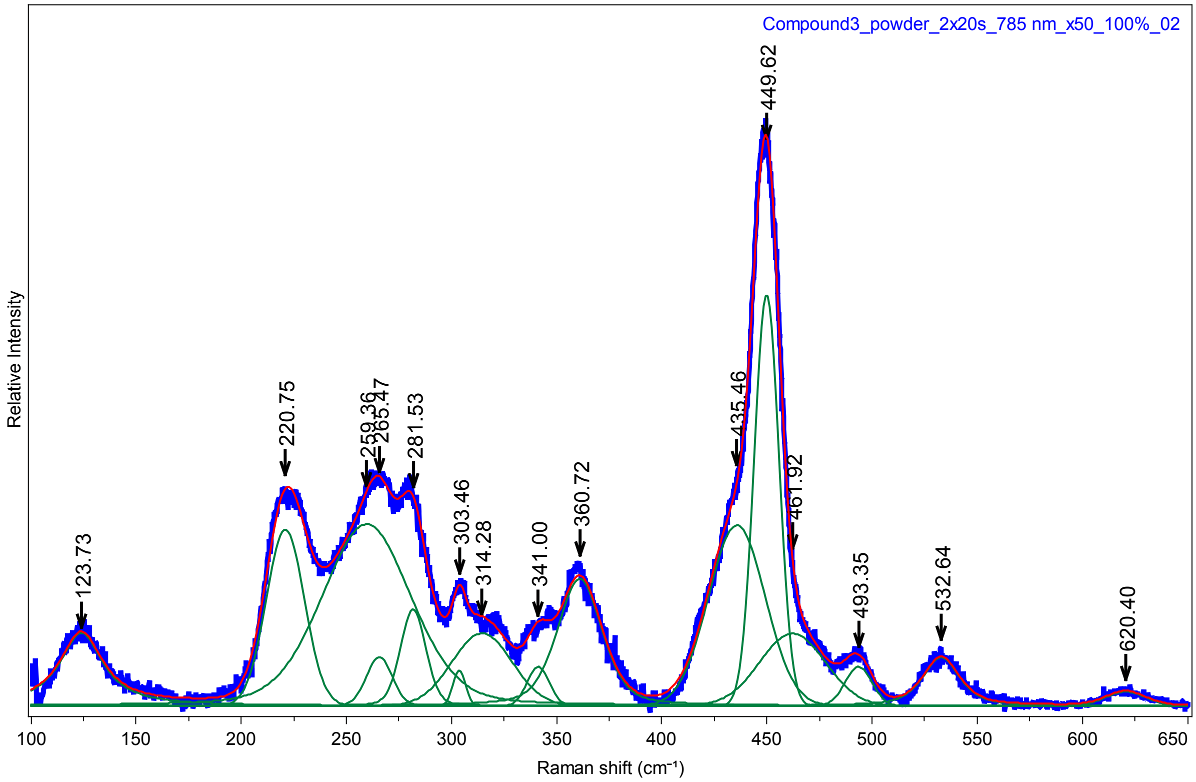

Figure S15 | Raman spectrum for a pure powder of **3** at room temperature. The data are presented in blue, the deconvoluted peaks are in green and the sum of the determined peaks is red, which overlays the experimental data.

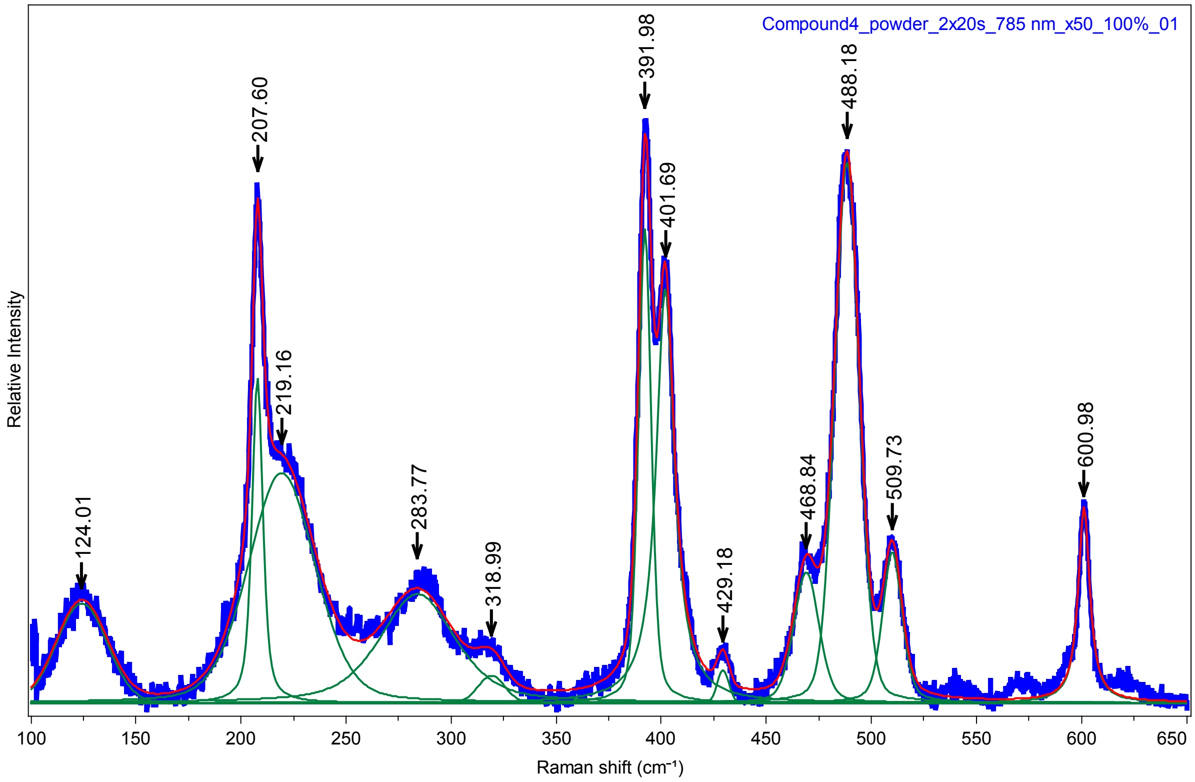

Figure S16 | Raman spectrum for a pure powder of **4** at room temperature. The data are presented in blue, the deconvoluted peaks are in green and the sum of the determined peaks is red, which overlays the experimental data.

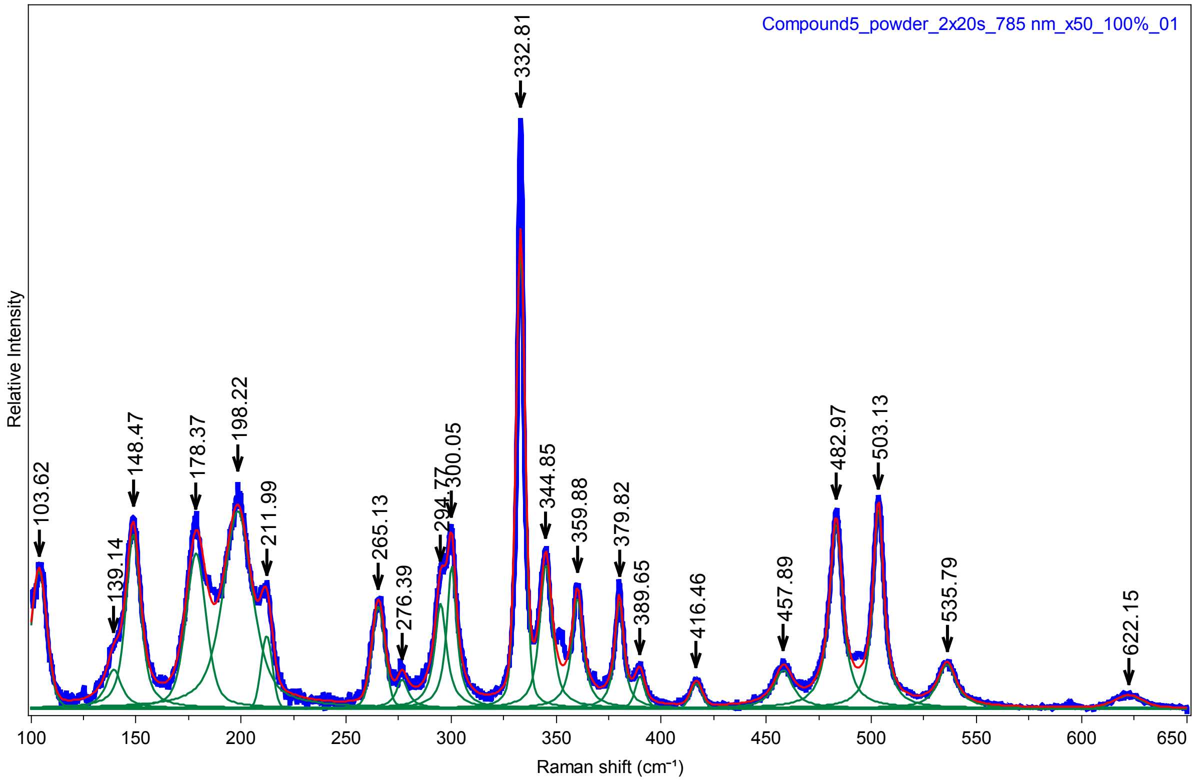

Figure S17 | Raman spectrum for a pure powder of **5** at room temperature. The data are presented in blue, the deconvoluted peaks are in green and the sum of the determined peaks is red, which overlays the experimental data.

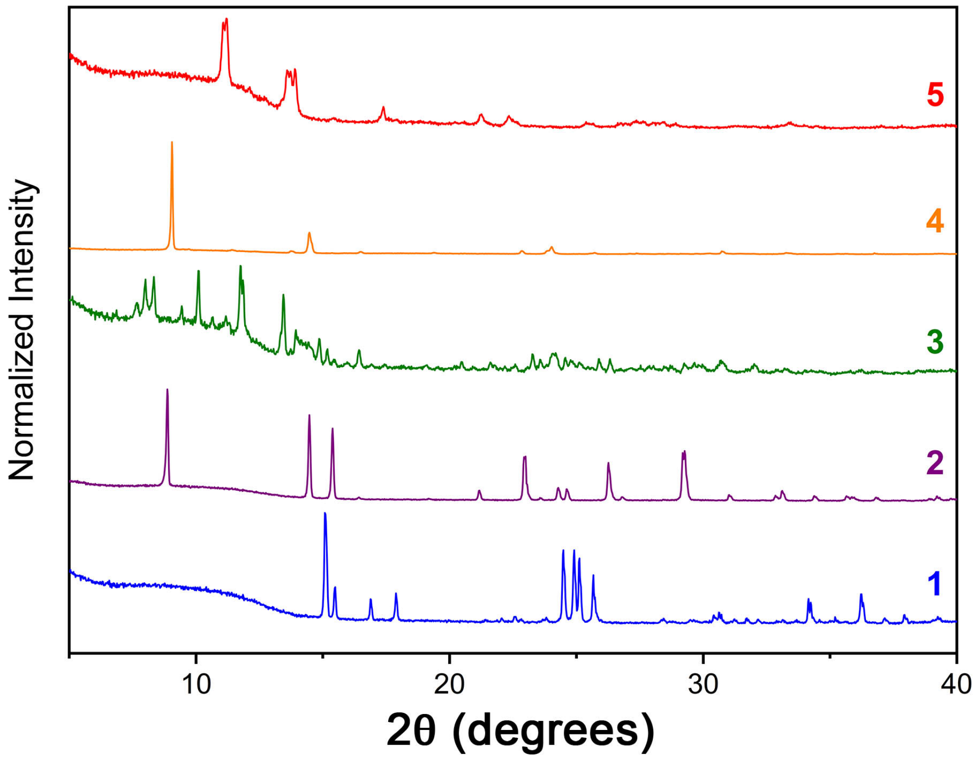

Figure S18 | X-ray powder diffraction data for **1-5**. Diffraction patterns were normalized to the highest intensity peak and offset prior to depiction. The low-angle humps are from the Vaseline used to restrict sample motion in the instrument.

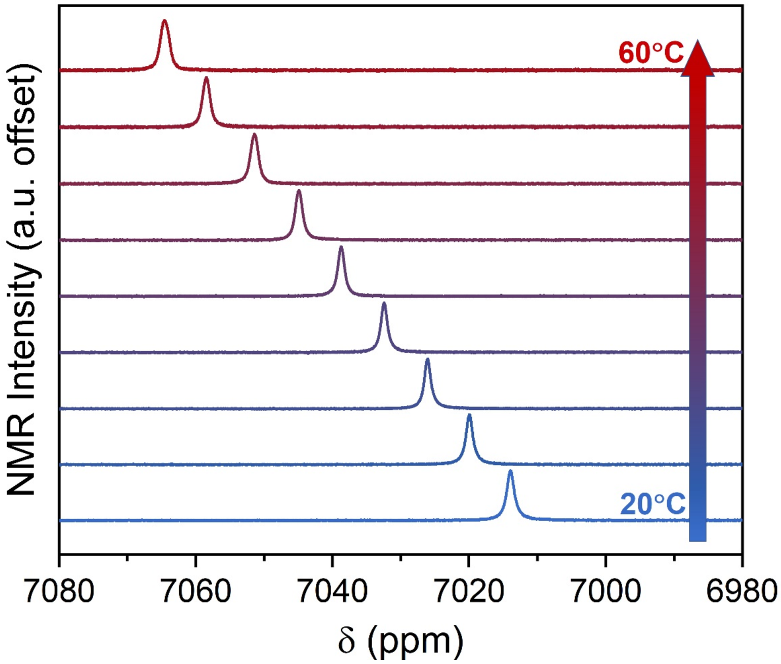

Figure S19 | Chemical shift data of compound **2** from 20-60 °C. NMR measurements were taken in a solution of DMSO at a concentration of ~20 mM.

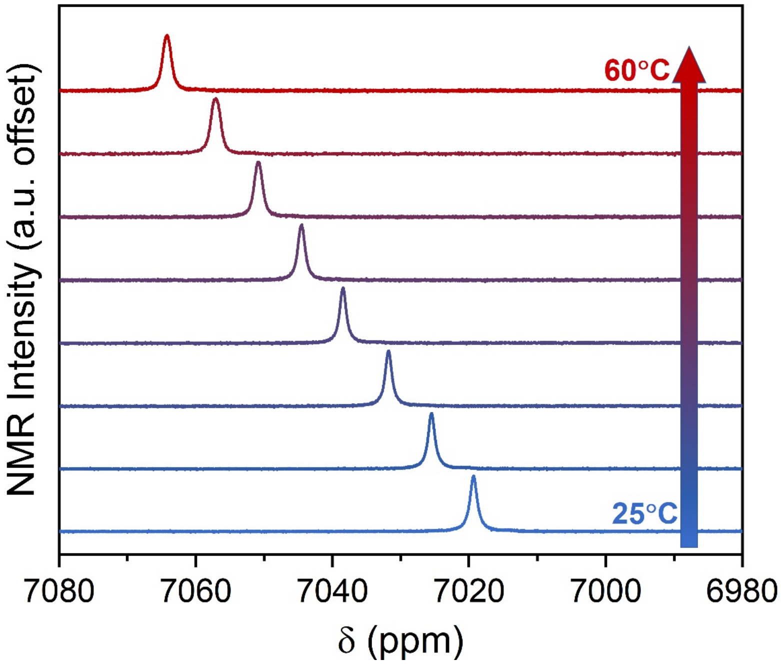

Figure S20 | Chemical shift data of compound **2** from 25-60 °C. NMR measurements were taken in a solution of d_6-DMSO at a concentration of ~20 mM.

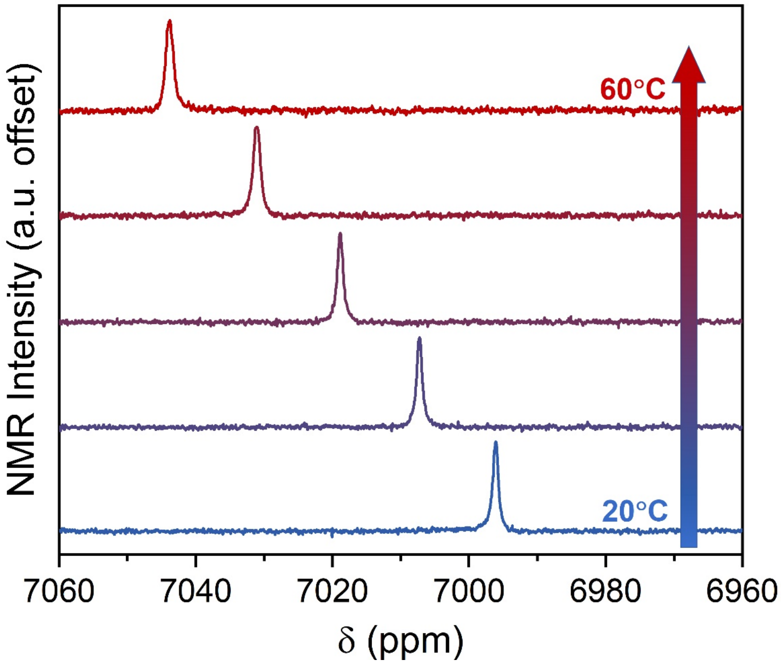

Figure S21 | Chemical shift data of compound **2** from 20-60 °C. NMR measurements were taken in a solution of DMF at a concentration of ~15 mM.

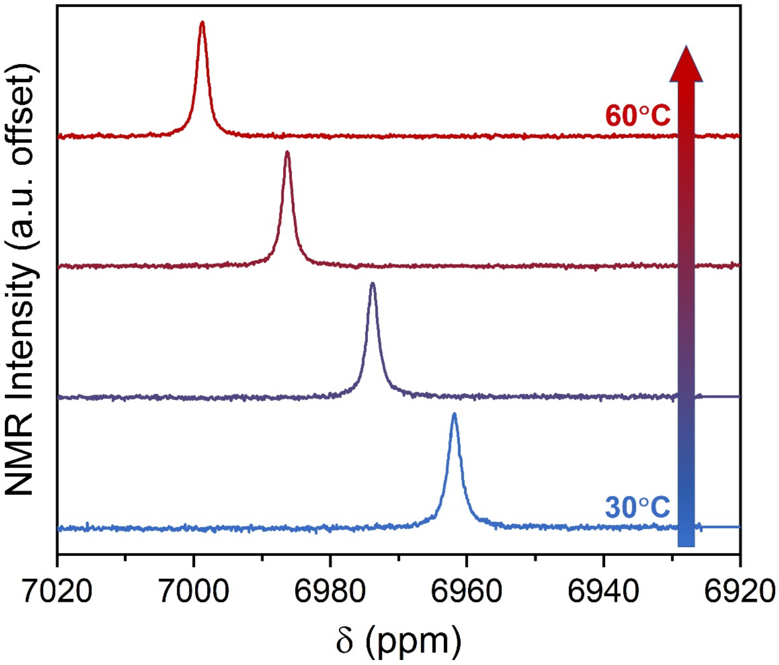

Figure S22 | Chemical shift data of compound **2** from 30-60 °C. NMR measurements were taken in a solution of HMPA at a concentration of ~10 mM.

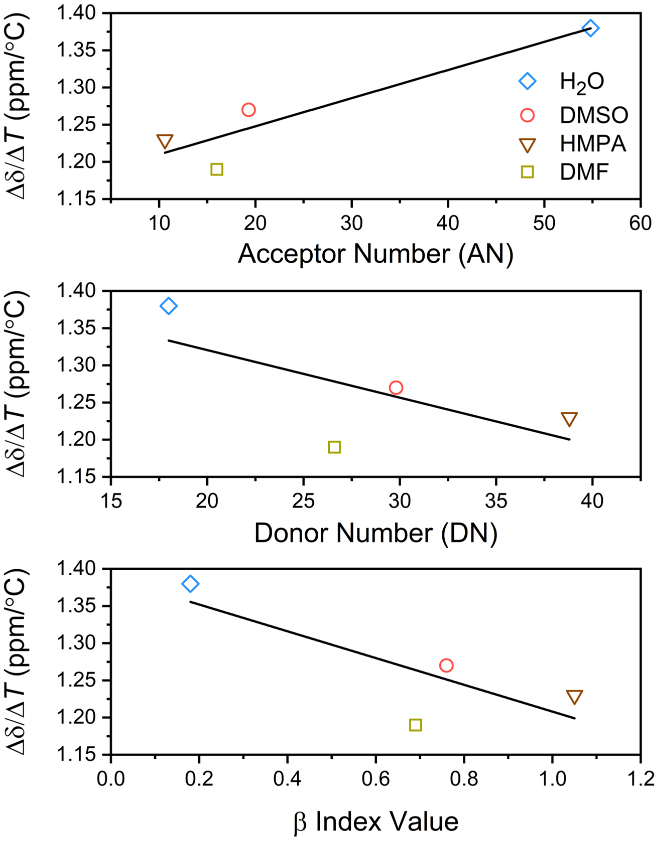

Figure S23 | Plots of [59]Co chemical shift sensitivity as a function of various solvent polarity indices for **2**. Acceptor/donor numbers for the utilized solvents were taken from ref 24, β values taken from ref 25. The solid lines are the results of linear regression (top: R^2 = 0.79; middle: R^2 = 0.18; bottom: R^2 = 0.45).

References

1 C.-J. Qin, L. James, J. D. Chartres, L. J. Alcock, K. J. Davis, A. C. Willis, A. M. Sargeson, P. V. Bernhardt and S. F. Ralph, *Inorg. Chem.*, 2011, **50**, 9131–9140.

2 R. A. Krause and E. A. Megargle, *J. Chem. Educ.*, 1976, **53**, 6268.

3 R. Bramley, M. Brorson, A. M. Sargeson and C. E. Schaeffer, *J. Am. Chem. Soc.*, 1985, **107**, 2780–2787.

4 J. C. Bailar and J. B. Work, *J. Am. Chem. Soc.*, 1946, **68**, 232–235.

5 R. J. Geue and M. R. Snow, *Inorg. Chem.*, 1977, **16**, 231–241.

6 H. Cai, J. Fissekis and P. S. Conti, *Dalt. Trans.*, 2009, 5395.

7 R. J. Geue, T. W. Hambley, J. M. Harrowfield, A. M. Sargeson and M. R. Snow, *J. Am. Chem. Soc.*, 1984, **106**, 5478–5488.

8 *MestReNova*, Mestrelab Research S. L., Escondido, CA, 2018.

9 *Origin*, OriginLab, Northampton, MA, 2018.

10 A. Buades, B. Coll and J. M. Morel, *Multiscale Model. Simul.*, 2005, **4**, 490–530.

11 J. V. Manjón, P. Coupé, L. Martí-Bonmatí, D. L. Collins and M. Robles, *J. Magn. Reson. Imaging*, 2010, **31**, 192–203.

12 Y. Masuda and H. Yamatera, *J. Phys. Chem.*, 1988, **92**, 2067–2071.

13 D. G. Gillies, L. H. Sutcliffe and A. J. Williams, *Magn. Reson. Chem.*, 2002, **40**, 57–64.

14 M. Kanakubo, H. Ikeuchi and G. P. Satô, *J. Magn. Reson. Ser. A*, 1995, **112**, 13–16.

15 M. Kanakubo, T. Uda, H. Ikeuchi and G. P. Satô, *J. Solution Chem.*, 1998, **27**, 645–653.

16 C. W. Kirby, C. M. Puranda and W. P. Power, *J. Phys. Chem.*, 1996, **100**, 14618–14624.

17 N. Galamba, *J. Phys. Chem. B*, 2013, **117**, 589–601.

18 J. D. Smith, R. J. Saykally and P. L. Geissler, *J. Am. Chem. Soc.*, 2007, **129**, 13847–13856.

19 A. Tokmakoff and M. D. Fayer, *Acc. Chem. Res.*, 1995, **28**, 437–445.

20 J. J. Turner, *Coord. Chem. Rev.*, 2002, **230**, 213–224.

21 S. C. F. Au-Yeung and D. R. Eaton, *Can. J. Chem.*, 1983, **61**, 2431–2441.

22 S. C. Au-Yeung and D. R. Eaton, *J. Magn. Reson.*, 1983, **52**, 366–373.

23 L. R. Gahan and J. M. Harrowfield, *Polyhedron*, 2015, **94**, 1–51.

24 W. R. Fawcett, *J. Phys. Chem.*, 1993, **97**, 9540–9546.

25 M. J. Kamlet, J. L. M. Abboud, M. H. Abraham and R. W. Taft, *J. Org. Chem.*, 1983, **48**, 2877–2887.
